# Rechargeable Metasurfaces for Dynamic Color Display Based on a Compositional and Mechanical Dual-Altered Mechanism

**DOI:** 10.34133/2022/9828757

**Published:** 2022-10-19

**Authors:** Le Yang, Xiaorong Hong, Jiafang Li, Chang-Yin Ji, Yu Han, Shanshan Chen, Hanqing Jiang, Wei-Li Song, Hao-Sen Chen, Daining Fang

**Affiliations:** ^1^ Beijing Key Laboratory of Lightweight Multi-Functional Composite Materials and Structures, Institute of Advanced Structure Technology, Beijing Institute of Technology, Beijing 100081, China; ^2^ Key Lab of Advanced Optoelectronic Quantum Architecture and Measurement (Ministry of Education), Beijing Key Lab of Nanophotonics & Ultrafine Optoelectronic Systems, School of Physics, Beijing Institute of Technology, Beijing 100081, China; ^3^ School of Engineering, Westlake University, Hangzhou 310024, China

## Abstract

Dynamic color display can be realized by tunable optical metasurfaces based on the compositional or structural control. However, it is still a challenge to realize the efficient modulation by a single-field method. Here, we report a novel compositional and mechanical dual-altered rechargeable metasurface for reversible and broadband optical reconfiguration in both visible and near-infrared wavelength regions. By employing a simple fabrication and integration strategy, the continuous optical reconfiguration is manipulated through an electro-chemo-mechanical coupled process in a lithium ion battery, where lithiation and delithiation processes occur dynamically under a low electric voltage (≤1.5 V). By controlling the phase transformation from Si to Li _x_Si, both structural morphology and optical scattering could be rapidly and dramatically tailored within 30 s, exhibiting high-contrast colorization and decolorization in a large-area nanofilm and showing long cyclic stability. Significant wide-angle reconfiguration of high-resolution structural colors in bowtie metasurfaces is demonstrated from anomalous reflection. The results provide a multifield mechanism for reconfigurable photonic devices, and the new platform can be introduced to the multidimensional information encryption and storage.

## 1. Introduction

Structural colors are generated by scattering, diffraction, or dispersion of optical interference or resonance and take the advantages of long-term stability, sustainable production, high resolution, and color purity compared to the common colors generated by dyes and pigments [ 1– 6]. The dynamic structural colors have the strong potential to realize the high-color performance covering the entire visible range by adjusting a single-design nanostructure and have been potentially explored by utilizing the mechanical-based microstructure regulation or the physical-/chemical-/electrical-based compositional adjustment [ 7– 11]. For example, mechanical stretching has been widely used to regulate the micro- and nanostructures of advanced materials [ 12– 16]. The specifically prepared elastomeric substrates or matrixes, in combination with the state-of-the-art metasurface designs, have been successfully adopted to realize broadband optical modulation in both the visible [ 9, 17– 19] and near-infrared [ 20, 21] wavelength regions. However, due to the macroscopic mechanical control model, most of the schemes could only tune the global optical behaviors, while the nonuniform local deformation often leads to the distortion of the displayed images.

In comparison, compositional control provides a stable methodology to tune the structural colors via changing the optical parameters (such as the real part n and imaginary part k refractive index components) of the materials and can be realized by selective chemical reaction or physical phase transition [ 7, 8, 22]. For instance, the gas reaction controlled phase-change materials, such as magnesium (Mg) and TiO _2_, have been developed for the dynamic tuning of plasmonic structural colors via hydrogenation and dehydrogenation in the presence of hydrogen or oxygen, respectively [ 7, 8, 22– 24]. Hydrogel-based structural colors have also been realized via the multilayer structure, and the color modulation was mainly driven by the change of environmental humidity [ 25– 28]. Besides, electronic-based compositional control has aroused great interest due to its advantages of simplicity and accuracy, which enable versatile strategies such as physical phase change method [ 29– 32], electrochemical deposition [ 33– 35], floating solid-state thin films [ 33], and electrochromic materials [ 36– 38]. However, due to the limited tunable range of the optical parameters, the reported compositional control methods are challenging to simultaneously realize multiple functionalities, such as broadband operation, stable reconfiguration, wide-angle regulation, and facile integration, which form the barriers to realistic applications.

In this article, we demonstrate a compositionally and mechanically dual-altered rechargeable metasurface for wide-angle and broadband optical reconfiguration based on the well-developed and preciously controlled lithiation and delithiation mechanisms. By simply fixing planar nanostructures into a lithium ion battery (LIB) cell, the rechargeable metasurface is accomplished based on a fully electro-chemo-mechanical (ECM) coupled process, in which the high-volume deformation (with a maximum ratio of 300%) occurs based on the phase change of amorphous Si to Li _x_Si under various applied voltages [ 39– 41]. With a low-voltage control in the LIB (≤1.5 V), both structural geometries and material properties are reversibly tuned, providing an effective scheme for multifunctional optical reconfiguration. As a result, the high-contrast colorization and decolorization in a large-area continuous nanofilm, as well as the reversible reconfiguration of high-resolution structural colors, are successfully achieved. In comparison, the structures in this work can be readily designed with high-resolution features (scale down to tens of nanometers), desirable sample areas, and well-defined geometries. Moreover, here, the changes in refractive index and structural morphology (including thickness and width) greatly improve the control ability and bring more potential advantages for nanophotonic transformations, such as structured light, flat lenses, and topological photonics. Especially, the locally designable deformation of the structure, rather than simple material deposition, may bring more degrees of freedom to tune optical phase, polarization, and frequency. The demonstrated rechargeable metasurface provides an effective methodology of dynamic color manipulation by utilizing the electrochemical process, which paves the way toward versatile design and fabrication of reconfigurable photonic devices, anticounterfeiting and secure information encryption, etc.

## 2. Results

### 2.1. Operation Mechanism of the Compositionally and Mechanically Dual-Altered Metasurfaces

**Figure 1:**
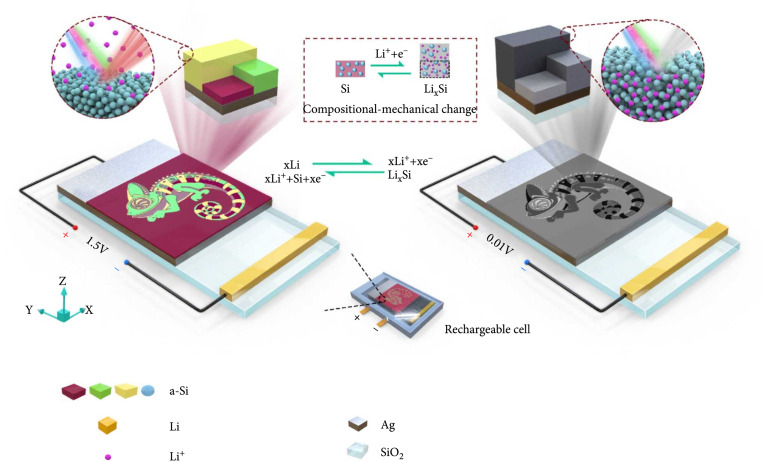
Compositionally and mechanically dual-altered metasurfaces based on an electro-chemo-mechanical process. The nanopattern is prepared by depositing Si of different thickness on an Ag layer coated on silica substrate, which shows colorful images (like the colorful chameleon) due to the thin-film interference. After integrated into a LIB cell, a potential is formed with a voltage of ~1.5 V (vs. Li/Li ^+^). After integrated into a LIB cell, a potential is formed with a voltage of ~1.5 V (vs. Li/Li ^+^). The classic Li metal is used as the counter electrode (and also as the role of reference electrode) in the half cell to avoid the influence of the various counter electrode materials. The chemical reaction equations are shown in the figure of the Li and Si electrodes. The half reaction for the Li metal reference electrode is xLi → xLi ^+^ + xe ^-^, and the half reaction for the Si electrode is Si + xLi ^+^ + xe ^-^ → Li _x_Si. By applying a constant current (150 *μ*A) to the cell, the Li ions are inserted into the Si film, which is driven by the electrochemical reactions. As a result, the color is erased due to the phase transition from Si to Li _x_Si, as the gray chameleon shown in the bottom-right. Such a process is reversible when the different voltages are applied; i.e., the colors can be turned on and off dynamically.

The fundamental mechanism of the rechargeable metasurfaces and corresponding color display is illustrated in Figure 1. The metasurface is firstly fabricated by patterning Si structures with thickness ( *d*) onto an Ag film coated on a quartz (SiO _2_) substrate (see Materials and Methods). Subsequently, the metasurface chip is assembled into a LIB, where the Ag layer serves as a current collector during the charging and discharging. Under the voltage control of the cell, the dynamic colorizing and decolorizing behaviors are tuned by the compositional change (i.e., Si to Li _x_Si, as revealed by the element distribution in Figure S3) and the mechanical change (i.e., volume expansion up to 300% [ 39, 40, 42]) of the Si during the discharging (lithiation by decreasing voltage to 0.01 V) and recharging (delithiation by increasing voltage to 1.5 V) processes, respectively (see Supporting Note S1 and the simulation results in Figure S4). Besides, combining the commercial counter electrode, the rechargeable metasurface system can also work as a lithium ion battery (LIB) with high capacity. The capacity of the LIB depends on both of the negative and positive electrodes in the battery. The capacity of Si electrode, which is our rechargeable metasurface, is about 3000 mAh g ^-1^, which is 10-fold higher than that of commercial graphite negative electrode [ 43]. However, in our experiments, the classic Li metal is used as the counter electrode (and also as the role of reference electrode) in the half cell to avoid the influence of the various positive electrode materials.

During lithiation, insertion of Li ions into Si layer could dramatically modify the optical response of the metasurfaces. First, the thickness of the Si layer increased, and its refractive index could be modified, which would change the optical path and consequently alter the thin-film interference. Second, the inserted of Li ions could change the spacing between the Si atoms and generate compositional inhomogeneity in the films. Such variations at the atomic scale could cause topographic changes, such as the increased surface roughness [ 44], which will introduce additional scattering and absorption during the lithiation process. More importantly, these two effects are dynamically reversible during the lithiation and delithiation processes, providing the basic operation mechanism of optical reconfiguration in the as-constructed rechargeable metasurfaces.

**Figure 2:**
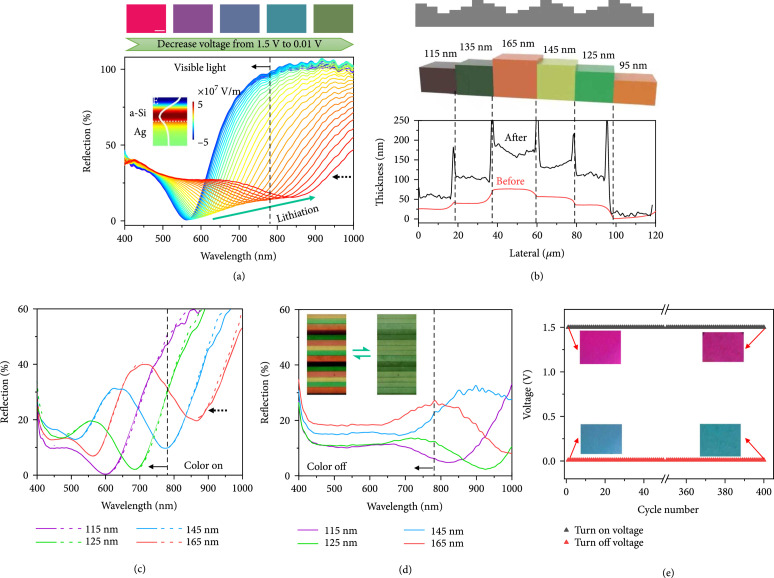
Tunable broadband thin-film interference. (a) Measured spectral evolution of a 105 nm thick Si film and the optical images at typical states by a home-build in situ electrochemical-spectrograph system (see Materials and Methods and Figure S1, Supporting Information) Scale bar: 50 *μ*m. CIE 1931 chromaticity diagram of the measured reflection spectra in (c) and (d) under the color “on” and “off” states is presented in Figure S8e in Supporting Information. This color regulation process can be completed within 30 s under constant potential conditions (Figure S2c, Supporting Information). Inset, E-field distribution of the initial state at interference wavelength λ=550 nm. (b) Designed stepwise Si nanostructure (top), correspondingly measured colors (middle), and the associated profile of film thickness before and after lithiation (bottom). (c) Reflection spectra of Si ribbons with different thickness under the color “on” state ( v=1.5 V) before (solid line) and after (dash line) a whole electrochemical cycle. (d) Reflection spectra of the Si ribbons under the color “off” state ( v=0.01 V). Inset, microscopy images of the Si ribbons with different thickness when the colors are reversibly turned on and off. Scale bar: 50 *μ*m. (e) The cyclic test of the metasurface. The fast charging/discharging tests were conducted under the constant voltage control. The cutoff voltage of the “on” and “off” states is set as 1.5 V and 0.01 V, respectively. The result shows the color stabilities of the metasurface after 400 cycles, in which the colors of the “on” and “off” states at 400th cycle are consistent with that in the first cycle.

Thin-film architectures are the basis of many important technologies such as semiconductor devices, optical coating, solar cells, and battery units, in which the reversible modification of thin-film properties is significant while it is still a great challenge. Here, the electrochemically reconfigurable optical properties of the nanofilms were readily demonstrated by adopting the lithiation and delithiation associated changes in film thickness and properties. As the experimental curves plotted in Figure 2(a), the reflection spectrum of a 105 nm thick Si film changes dramatically during the lithiation progress, in which the dip wavelength $\lambda_{d}$ is red-shifted from 560 to 840 nm, corresponding to a wavelength shift of $∆\lambda_{d}/\lambda_{d}=50\%$ . Such reflection dip is induced by the thin-film interference (see inset of Figure 2(a) for the interfered electric field distribution), and the wavelength was determined by $\lambda_{d}=Nn_{\text{eff}}d/2$ , where N=1,2,3⋯ and $n_{\text{eff}}$ is the effective refractive index of the Si film (see Figure S2b, Supporting Information). This broadband tuning of the interference wavelength is partially caused by the increased film thickness (increased d ) during the lithiation process, which is confirmed by the in situ atomic force microscope (AFM) results in Figure S2 that quantitatively reveal the large deformation during the phase change from Si to Li _x_Si of the metasurface. The initial thickness of the Si film measured by AFM is about 110 nm, which is consistent with the result measured by a step profiler (Figure S2, Supporting Information). After the lithiation process, the film thickness increases to around 300 nm, which means the thickness of the film is increased by around 190 nm during the phase change from Si to Li _x_Si.

The basic color evolution in Figure 2(a) (see continuous color changes in Supplementary Movie S1) provides great feasibility for dynamic color printing by engineering the film thickness. To this end, a stepwise Si ribbon structure is designed (top of Figure 2(b)) and fabricated by repeated ultraviolet exposure and film deposition processes (see Materials and Methods and Figure S6, Supporting Information). As shown in the middle of Figure 2(b), six different colors from Si ribbons of various thickness were clearly observed in the experiments. The thicknesses of the switched Si ribbons were measured as shown in the bottom of Figure 2(b), in which one can also find the remarkable swell of the film after the electrical treatment. The deformation of the films during the lithiation process under electrochemical loading forms the foundation of the mechanical modulation. We also analysed the root-mean-square (RMS) of the rough data from x=0 *μ*m to x=15 *μ*m in Figure 2(b), which is the first stage, and the results show that the RMS of roughness is 0.40 and 11.47 nm ^2^ before and after lithiation, respectively. The corresponding spectral measurements in Figure 2(c) show clear film interference features along with a linear variation of dip wavelength when the film thickness changes (Figure S7b, Supporting Information). Importantly, the multiple colors can be simultaneously and reversibly tuned by the cyclic lithiation and delithiation processes, as shown by the images in the inset of Figure 2(d) and the spectra in Figures 2(c) and 2(d) (see continuous color changes and spectral evolution in Supplementary Movie S2 and Figure S8, Supporting Information, respectively). Another interesting observation is that all the reflection dips shift outside the visible wavelength region when the lithiation is fully implemented. Therefore, the overall colorful ribbons were turned into ribbons with nearly the same colors (green or dark green), as shown by the simultaneous color switching of the ribbons in the inset of Figure 2(d). A CIE 1931 chromaticity diagram of the measured reflection spectra in Figures 2(c) and 2(d) under the color “on” and “off” states is shown in Figure S8e in Supporting Information. We can see the changes from colorized state to decolorized state due to the shifts of the chromaticity coordinates. In such a case, the ribbons can be dynamically and reversibly colorized and decolorized through cyclic lithiation and delithiation processes.

Importantly, such a broadband tuning of the spectra results in a dramatic change of the thin-film colors and can be accomplished within 30 s under constant potential conditions (Figure 2(e)). The fast charging/discharging tests were conducted under the constant voltage control. The cutoff voltage of the “on” and “off” states is set as 1.5 V and 0.01 V, respectively. The result shows excellent color stabilities of the metasurface after 400 cycles, in which the colors of the “on” and “off” states at 400th cycle are consistent with that in the first cycle. The corresponding time-resolved color change of the metasurface (as shown in Figure S5a, b, Supporting Information) reveals that the color regulation can be consistently accomplished within 30 s (equivalent to 120 C charge rate). For a firmly sealed battery, its lifetime is typically given as the number of charge/discharge cycles. Thus, the rechargeable metasurfaces can possess at least 400 cycles life span at 120 C high charge rate. Besides, the HSL (hue, saturation, and lightness represent the color, the intensity or purity of a hue, and the relative degree of black or white, respectively) results of the cyclic test are shown in Figure S5c-e in Supporting Information.

Note that the broadband decolorizing effect is caused by both the increase of film thickness (i.e., mechanically) and induced scattering and absorption during the lithiation (i.e., compositionally). This is because the wavelength position of the spectral dip is determined by the thin-film interference. As a result, with the increase of the film thickness after lithiation, the spectra of the lithiated 105 nm thick Si film (the rightmost line in Figure 2(a)) show the similar dip position around 860 nm as that of the initial Si film with thickness of 165 nm (red line in Figure 2(c)). In comparison, at the end of the lithiation process, the interference dip is nearly invisible, and the spectrum shows a flat-band response in Figures 2(a) and 2(d), which are mainly induced by the change in material properties (such as the real and imaginary parts of the refractive index of the materials, referenced in our previous investigation [ 45]) based on the compositional mechanisms. Therefore, the mechanical deformation of the film dominates the position change of the spectral dip, which mainly determines the color change at the initial stage of lithiation. In comparison, the compositional change of the Si to Li _x_Si mainly contributes to the broadband absorption of the metasurfaces at the end of lithiation. As a result, the final films with different initial thickness possess the similar broadband spectra in the visible wavelengths, and they all show similar dark green colors after the lithiation, as illustrated by the reflection spectra in Figure 2(d).

### 2.2. Dynamic Display of the Chameleon and Butterfly

**Figure 3:**
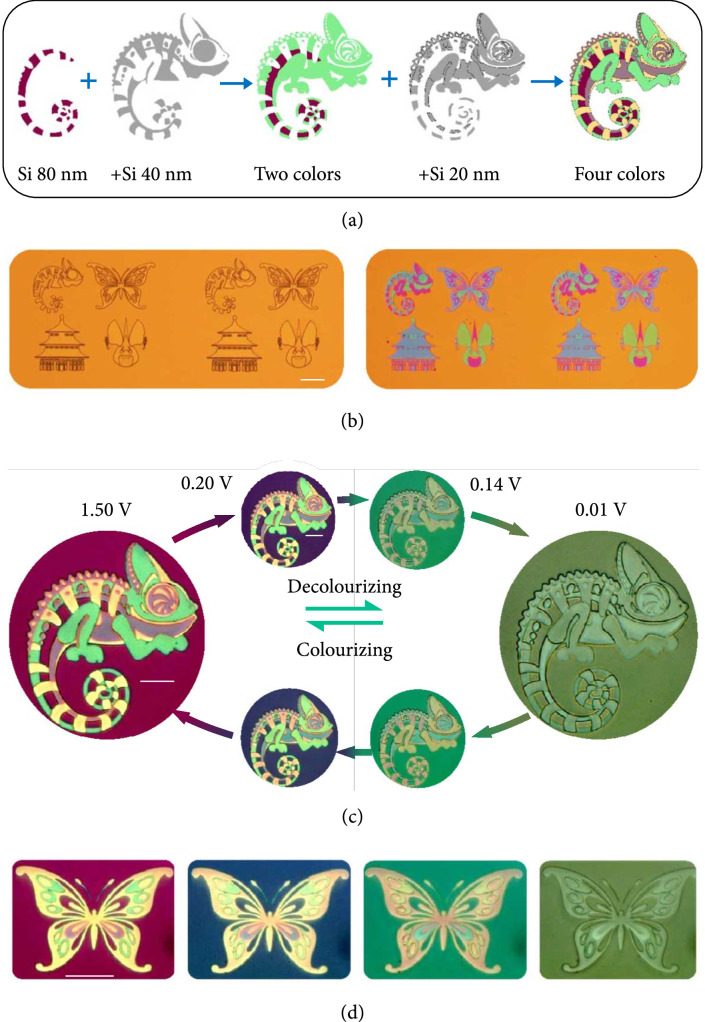
Dynamic color decolorizing and colorizing. (a) Schematic of the design and fabrication of a colorful chameleon. Four colors can be displayed by repeating the laser exposure and film deposition process on the 80 nm thick Si film. (b) As-recorded dark-field optical microscopy images of the large-area fabricated patterns before and after the complete Si deposition (Figure S10, Supporting Information). The sample size of four patterns is 200μm×200μm . Scale bars: 50 *μ*m. (c, d) Optical microscopy images of the fabricated chameleon and butterfly during reversible colorizing and decolorizing processes. Benefited from the stable states of the Li _x_Si, every intermediate color could be maintained when the voltage is off. Scale bars: 20 *μ*m.

The polychromatic colors of the rechargeable stepwise ribbons inspire a new strategy for electrically dynamic color printing, promising a novel technology in color encoding and anticounterfeiting and secure information encryption [ 27, 28]. To demonstrate this feature, a chameleon-like pattern was prepared by a multilayer film deposition method in Figure 3(a) (see Materials and Methods). Specifically, two photomasks were employed for laser exposure and a subsequent film deposition process (see details in Figure S9a, Supporting Information), which resulted in a chameleon pattern with Si layers of four thickness (80, 100, 120, and 140 nm). Such a fabrication method is very satisfying for large-scale color printing, such as the eight patterns formed during the fabrication process in Figure 3(b) (see Figure S10 in Supporting Information for large patterns with size over 1.5×1.5 mm ^2^). The as-fabricated film patterns were then integrated into the battery chamber, followed by electrochemically activating by a pre-charge-discharge cycle. Finally, the stabilized film patterns in the LIB cell show four different colors (dark violet, green, yellow, and light violet), as the microscopy images of “chameleon” and “butterfly” vividly shown in Figures 3(c) and 3(d) (see more images in Figure S9b, Supporting Information).

One important feature of our rechargeable schemes is that the reversible colorization and decolorization of chameleon and the butterfly can be readily achieved by simply controlling the external voltage within 1.5 V, as shown by the optical images in Figures 3(c) and 3(d) (see continuous color evolution in Supplementary Movie S3 and S4). After the complete lithiation, all the initial colors were found to change to the similar dark green color to the background, i.e. “the chameleon’s skin replicates the color of its surroundings.” When the delithiation process was completed, the colors of chameleon and butterfly were fully recovered to the initial state. It should be mentioned that the optical response of the metasurfaces could be simply determined by the amount of the inserted Li ions, which is directly associated with the electrochemical potential of the Li _x_Si. As a result, the structural colors are highly correlated with the applied voltage. Therefore, every intermediate color during the continuous evolution processes could be maintained (settled) at the certain states even after removal of electrical connection, as the different colorful images shown in Figures 3(c) and 3(d) and Figure S9b in Supporting Information. Such a significant and desirable color manipulation strategy under low-voltage operation provides a new method for reconfigurable metasurfaces.

### 2.3. Reconfigurable Optical Metasurfaces

**Figure 4:**
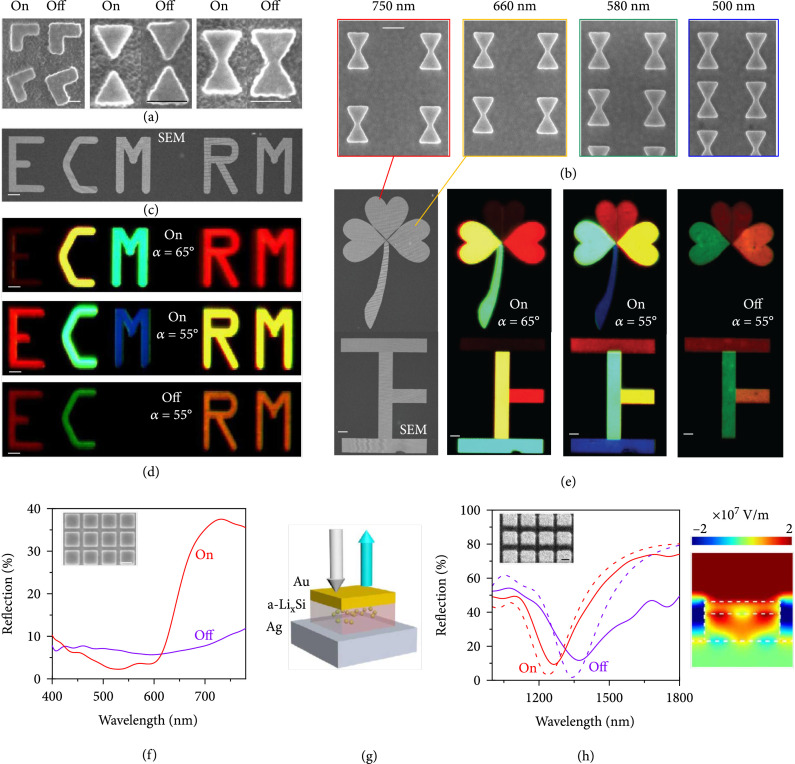
Reconfigurable nanostructures and structural colors. (a) SEM images of typical L-shaped, separated bowtie, and touched bowtie nanostructures before (“on” state) and after (“off” state) the lithiation. (b) Top-view SEM images of bowtie nanostructures with different period as noted. (c) SEM image and (d) optical micrographs (at $\alpha =55^{{}^{\circ}}/65^{{}^{\circ}}$ and $\beta =10^{{}^{\circ}}$ ) of the decrypted (“on” state) and encrypted (“off” state) bowtie nanostructures with different period. The letter “E” can be crypted by changing the incident angle, and the letter “M” can be crypted by turning off the rechargeable metasurfaces. (e) SEM images and optical images of the “clover” and a Chinese characters “王” (denote “huge”) at the “on” and “off” states. The petiole of the “clover” is hidden, and the “王” changes to another Chinese character “下” (denote “down”) at the “off” state. The character “王” changes to another Chinese characters “上” (denotes “up”) when α increases from 55° to 65°. (f) Reflection spectra of a square array of Si squares with d=280 nm and p=380 nm under normal incidence. Inset, SEM image of the Si squares. (g) A FP-like nanoresonator. Structure parameters: d=400 nm, p=500 nm, and thickness of the top gold layer t=47 nm. (h) Reversible reflection spectra of the nanoresonator. Top inset, SEM image of the square array. Right inset, calculated *E*-field distribution at the dip wavelength (1240 nm) of the simulated reflection spectrum (dash line). Scale bar: 200 nm in (a), (b), (f), and (h) and 20 *μ*m in (c)–(e).

The fabrication resolution of the laser exposure and film deposition method could be limited by both the feature size of the photomask and the wavelength of the laser. To demonstrate the high-resolution structural colors in the rechargeable metasurfaces, the electron beam lithography (EBL) method was further adopted for the pattern exposure, and inductively coupled plasma (ICP) etching processes were used to transfer the nanopatterns into the 105 nm thick Si film (Figure S11, Supporting Information). For general illustrations, the widely studied L-shaped metasurfaces and bowtie nanostructures with different periods and gaps are fabricated in Figure 4(a) (see more details in Figures S12 and S13, Supporting Information). The contact area of nanostructured Si between the Si surface and electrolyte is larger comparing with that of the Si film due to the increase of the side area of the Si nanostructures. Thus, its lithiation path is shorter than Si film. Upon lithiation, the morphologies of the nanostructures changed notably, which reveals a lateral deformation of ~20 nm, consistent with the simulation results in Figure S14. The lateral deformation under lithiation was more obvious in the bowtie nanostructure, and its gap decreased from ~50 to ~20 nm along with tips nearly connected. Such a controllable nanoscale deformation is potential in the manipulation of geometrical morphologies, such as the controlling of ultrasensitive nanogaps for quantum plasmonics [ 46].

To study the modulations of high-resolution structural colors by the insertion of Li ions, L-shaped and bowtie nanostructures with various configurations were integrated into the LIB chamber and in situ characterized by a home-built scattering microscopy system. Although both L-shaped and bowtie nanostructure arrays showed very different colors and intensity modulations before and after the lithiation process (Figure S10, Supporting Information), the connected bowtie nanostructures possessed the highest reflection intensity among all the metasurfaces (Figure S13c, Supporting Information). Based on such basic units, the structural colors in Figures 4(b)– 4(e) were simply designed based on the diffraction equation $p\times \left(\sin\alpha -\sin\beta \right)=j\lambda$ , where p is the period of the structures, λ the wavelength, j the diffraction orders, and $\alpha \left(\beta \right)$ is the incident (reflection) angle. In such a case, the structural colors can be generated by changing the period of the structures. For example, the values of p=750 , 660, 580, and 500 nm were selected in Figure 4(b), and thus, high-resolution “blue,” “yellow,” “green,” and “blue” colors could be generated in the oblique diffraction at $\alpha =55^{{}^{\circ}}$ and $\beta =10^{{}^{\circ}}$ (as shown in Figures 4(d) and 4(e)). Here, the minimum pixel size is 500 nm × 500 nm, which is determined by the fabrication accuracy and the material absorption. If we use a different N. A. objective or the samples are tilted, the colors of Si-film will be different due to the grating diffraction.

As shown in Figures 4(d) and 4(e), the characters and patterns can readily be constructed by patterning bowtie nanostructures with different periods, which resulted in colorful characters and patterns. The background of the color patterns in Figures 4(d) and 4(e) is dark, because the incident angle of the light and the detection angle of the lens are aligned in certain diffraction angle. In this case, only the color patterns can be seen, but the unstructured areas are dark due to the unequal incident and reflection angles. It is found that the initial colors of the structure can be varied by changing the incident and observation angle. For example, the initial characters “ECM RM” “王” change to “CM RM” and another Chinese character “上” (denotes “up”), respectively, by simply changing α from 55° to 65°. Such angle-dependent mechanism provides an interesting freedom for the multidimensional information encryption. More importantly, both the structural colors and the intensity could be dynamically modulated by the electro-chemo-mechanical coupled lithiation and delithiation process. For instance, by simply applying external voltage bias, the colorful characters of ECM, RM, and 王 (i.e., “huge” in Chinese) could be change to EC, RM, and 下 (i.e., “down” in Chinese), respectively. This voltage-dependent reconfiguration provides a more advanced methodology for information encryption. Such a proof-of-concept demonstration not only proves the modulation capability of high-resolution structural colors in the rechargeable metasurfaces but also promises potentials for information encryption and anticounterfeiting applications.

The rechargeable metasurfaces can also serve as a platform for the building up of hybrid nanostructures by adopting the Si nanostructures as the substrate. To verify the feasibility, an array of square Si pillars (as the substrate) was firstly patterned on the Ag film (inset of Figure 4(f)), and the refection peak was red shifted with an intensity drop by more than 70% after the lithiation. Moreover, both the position and intensity of such modulation can be tailored by varying the pillar width (see Figures S15 and S16, Supporting Information), indicating a flexible design of the reconfigurable nanostructures. With such nanostructures, a metal-dielectric-metal (MIM) Fabry-Pérot (FP) cavity could be adopted for color displays [ 47, 48] via adding a 47 nm thick Au layer on the top of the Si pillars (Figure 4(g)). Although the Au layer will restrict the lithiation path at the top surface of the Si, the side surfaces are employed to offer the channel for Li insertion since the maximum lateral dimensions of the square pillars are only 400 nm. This mechanism is clearly proved by the simulation results in Figure S17 that the Li is inserted into the Si from the side surfaces, and the Si can be fully lithiated. This hybrid design brings up an important feature; i.e., the cavity length can be dynamically tuned by the lithiation and delithiation processes, which is very promising by adopting the grayscale patterned FP cavity [ 47, 48]. Due to the increase of cavity length caused by the insertion of Li ions, the initial resonant reflection dip at 1240 nm was shifted to 1390 nm after the lithiation, as plotted in Figure 4(h). The modulation was well consistent with the simulation results, where a localized FP mode could be formed within the FP cavity (right inset of Figure 4(h)). It should be mentioned that at long wavelength region, the increase of the refractive index of the material and the ratio between the increased thickness to the operation wavelength are less significant than those at shorter wavelengths. Although preliminary, such a demonstration is promising for future integration with versatile stepwise cavity resonators and application towards advanced metasurface designs, such as reconfigurable polarization converter [ 49, 50] and optical vortex generation [ 51]. Besides, this demonstration also shows that comparing to the depositional electrochemical control mode [ 34], the stably insertional electrochemical control mode could be appropriate for metasurfaces with more complex structures and can be used to realize more broadband optical reconfiguration.

## 3. Discussion

In summary, we have designed and fabricated a series of compositionally and mechanically dual-altered rechargeable metasurfaces based on the ECM-induced phase transformation from Si to Li _x_Si. By simply integrating the metasurfaces in a LIB chamber, the lithiation and delithiation processes were dynamically altered within a low cost voltage window of 0.01 V-1.5 V, which induced dramatic change in both mechanical structural thickness and compositional material properties. As a result, high-contrast colorizing and decolorizing in broadband visible wavelengths could be achieved in the continuous large-area nanofilm patterns. High-resolution structural colors in bowtie metasurfaces, as well as the tailorable MIM FP cavity, have revealed potentials for the wide-angle reconfigurable metasurfaces via nanostructure design or functionality hybridization. Such high-resolution structural colors are desirable for information encryption and storage. For example, based on the advantage of the convenient electronic control, the pixelated electrodes could be established in the future, which are very desirable for more complex functions and reconfigurations. Moreover, the advantages of our method, i.e., long-time stability, multifunctional integration, and facile production (considering the well-developed and continuously innovated battery technology and industry), could also be extended with some strategies of all-solid-state electrochromic devices [ 52, 53]. That is, developing all-solid-state rechargeable metasurface structural coloration could be a potential strategy in the future once a suitable solid-state material is applicable to replace the electrolyte. Besides, in order to enhance the switching rate of the rechargeable metasurface, the higher voltage range and applied current could enhance the switching rate in a certain limit. The rate determining step of the rechargeable metasurface is the reaction rate between the Li ions and the Si material at the lithiation front inside the film. The polarization at the surface of the electrode will result in the instability increase of the system if the electrochemical load is beyond the limit. Thus, a balance point should be found in the actual application taking into account of both the switching rate and durability of the metasurfaces. The cycling stability of the thin film Si electrode under 100 nm is about 1000 times .

In addition to the dynamic color display, the rechargeable metasurfaces have great potentials to realize dynamic control of light phase and polarization. For example, an optical vortex converter can be envisioned with the flexible design and fabrication strategies (see more details in Figure S18, Supporting Information), which can be used to achieve customized modulations of beam orbital angular momentum by charging and discharging different phase regions in the future. Furthermore, this rechargeable metasurface could play an important role in topological photonics. For instance, the morphology of quasi-2D and 3D topological photonic crystals in optical wavelengths can be changed via charging and discharging to realize customized reversible regulation of topological angle states, edge states, or topological transport, which could empower the emerging topological photonics with dynamic tunability [ 54]. Therefore, our rechargeable metasurfaces have built up a solid platform towards facile integration and effective configuration of metasurfaces and other photonic structures, which suggests a promising stage of applications in optical reconfigurations, dynamic color printing, security tagging, structural cryptography, etc.

## 4. Materials and Methods

### 4.1. Numerical Simulations

Numerical simulations were carried out by using commercial software COMSOL Multiphysics based on a finite element method. Periodic boundary conditions and waveguide port boundary conditions were used for calculation of the nanostructure arrays. Perfectly matched layers and background field conditions were used for calculations of the single structures. The simulations were carried out with the substrate.

### 4.2. Sample Fabrications

The quartz wafers (300 *μ*m thick, double-side polished) were used as substrates for metasurface fabrications. The current collector layer (Ag) and metasurface layer (Si) were deposited onto the quartz substrate by using a commercial magnetron sputtering system (Kurt J. Lesker LAB 18). First, the SiO _2_ substrate was plasma-cleaned in Ar at RF power of 50 W and 10 mTorr pressure for 3 minutes. Next, a 10 nm Cr adhesion layer was sputtered on the substrate followed by deposition of ~100 nm thick Ag layer. Then, for the preparation of Si film used in Figure 2(a), another 10 nm Cr and silicon layer of various thickness were deposited. Finally, the silicon thickness was measured by step profiler after the deposition (Figure S2a, Supporting Information). For the multilayer Si stepwise structures (Figure S9, Supporting Information), the patterns of Si ribbons (with ribbon width of 120 *μ*m) were prepared based on the standard photolithography. As the first layer, a ~95 nm thick silicon was deposited. After that, the photoresist was lift off. The same pattern was photolithographed again with a lateral shift ~20 *μ*m. Then, the second layer ~20 nm Si was deposited. By repeating the same process, the third layer (~10 nm Si) and the fourth layer (~40 nm Si) were prepared and form the final stepwise structures with various thickness. During the photolithography processes of the “chameleon” and “butterfly” structures in Figure 3, the ultraviolet exposure with masks was replaced by laser direct writing exposure (DWL66+, Heidelberg), and the other deposition processes stay the same. The EBL process was conducted in Tianjin H-Chip Technology by the standard EBL procedure. A dual beam FIB/SEM system (FEI Helios G4 UC) was used to prepare the sample with Au layer on the top Si squares (Figure 4(g)). Besides, the deposited films needed to be activated before the optical characteristics, as shown in Figure 3. The colors of the patterns under normal incidence will change from Figure 3(b) to the stable reversible initial colors in the first image of Figure 3(c) after electrochemically activated by a pre-charge-discharge cycle. This activation is necessary due to the formation of irreversible solid electrolyte interphase (SEI) at the surface of Si during the first electrochemical cycles. Nevertheless, the SEI film is very stable during the subsequent electrochemical cycles [ 55, 56].

### 4.3. Optical Characterizations

The in situ spectroscopic system is shown in Figure S1, which includes a home-build in situ electrochemical cell, the voltage control devices, and a home-build spectroscopy system. The home-build in situ electrochemical cell, including the rechargeable metasurface, the reference electrode, the shell, and the optical window, was assembled in a high-purity argon filled glove box (Mbraun Inc.) with H _2_O and O _2_ contents<10 ppm. The electrolyte filling in the cell consisted of 1 M LiPF _6_ in 1 : 1 ( v/v ) DMC: EC. For reflection spectral measurement with a modified microscope (BX53, Olympus), visible white and infrared light are from a tungsten halogen source (HL-2000, Ocean Optics) and supercontinuum light source (SC-PRO, YSL Photonics), respectively. For the vertical reflection experiments, the light was focused onto the sample by an objective lens (×10, NA 0.3, Olympus), and the reflected light in was collected by the same objective and delivered to a spectrometer (PG2000pro, Ideaoptics for visible wavelengths and NIRQuest, Ocean Optics for near-infrared wavelengths). For the anomalous scattering spectral experiments, the incident light was controlled by the home-built optical path. For the in situ optical imaging, an optical microscopy (Keyence Corporation) was used to characterize the continuous color changes in situ through the optical window on the electrochemical cell. All the electrochemical loads during the experiments were conducted by using a voltage control system (Arbin Instruments). The galvanostatic current density was 300 *μ*Acm ^-2^ and 300 *μ*Acm ^-2^ for discharging and charging, and the cycled voltage range was run from 1.5 V to 0.01 V (vs. Li/Li ^+^). Under this constant current loading condition, the average power is ~0.3 mW/cm ^2^, and~0.2 mWh/cm ^2^ energy is needed during the total charge process. The active area (area involved in the electrochemical reaction) of metasurface chip used in this paper is 0.5 cm ^2^.

## Data Availability

The data that support the findings of this study are available within the article and its supplementary materials.

## Supplementary Materials

Supplementary Materials Supplementary Materials include the Supporting Note S1, Supporting Figures S1 to S19, and legends for Movies S1 to S4.
