# Supplementary material for: Rechargeable Metasurfaces for Dynamic Color Display Based on a Compositional and Mechanical Dual-Altered Mechanism
Source: Research (Wash D C). 2022 Oct 19;2022:9828757. doi: 10.34133/2022/9828757 (PMC11030115; doi:10.34133/2022/9828757)
Supplement: Supplementary Materials — include the Supporting Note S1, Supporting Figures S1 to S19, and legends for Movies S1 to S4. [file 9828757.f1.zip › 2.1-supplementary materials.docx]

**Supplementary Materials for**

Rechargeable Metasurfaces for Dynamic Color Display Based on a Compositional and Mechanical Dual-Altered Mechanism

Le Yang^1,^ †, Xiaorong Hong^2,^ †, Jiafang Li^2,^ *, Chang-Yin Ji^2^, Yu Han^2^, Shanshan Chen^2^, Hanqing Jiang^3^, Wei-Li Song^1,^ *, Hao-Sen Chen^1,^ *, and Daining Fang^1,^ *

^1^Beijing Key Laboratory of Lightweight Multi-Functional Composite Materials and Structures, Institute of Advanced Structure Technology, Beijing Institute of Technology, Beijing 100081, China

^2^Key Lab of Advanced Optoelectronic Quantum Architecture and Measurement (Ministry of Education), Beijing Key Lab of Nanophotonics & Ultrafine Optoelectronic Systems, and School of Physics, Beijing Institute of Technology, Beijing 100081, China

^3^School of Engineering, Westlake University, Hangzhou 310024, China

*Corresponding authors. E-mails: jiafangli@bit.edu.cn; weilis@bit.edu.cn; chenhs@bit.edu.cn; fangdn@pku.edu.cn

†These authors contributed equally to this work.

**Supplementary Materials include:**

Supporting Note S1

Supporting Figures S1 to S19

Legends for Movies S1 to S4

Supplementary References

**Other supplementary materials for this manuscript include the following:**

Movies S1 to S4

**Supporting Note S1**

**Compositional and mechanical dual-process in the metasurfaces**

Based on electrochemical reaction process shown in Figure 1, lithium atoms in the counter electrode (Li metal) are ionized and separated from their electrons during the discharging process of the battery, which generates Li ions that subsequently migrate into the electrolyte. Consequently, Li ions capture the electrons at the surface of the metasurface and then insert into the Si film. The maximum ratio of Li: Si in the Si electrode could reach 3.7:1 in molar weight at room temperature, equivalent to a volume expansion more than 300%. In principle, the deformation gradient tensor ***F*** of Li_x_Si during lithiation could be decomposed into elasticity, plasticity and concentration parts, i.e. ***F=F_e_*•*F_p_*•*F_c_***. The corresponding principal stretches can be decomposed into three parts, i.e. *κ_i_=κ_i_^e^κ_i_^p^κ_i_^c^,* where *i=x,y,z,* denotes the directions*.* During the lithiation process, the elastic deformation is much smaller than the plastic and concentrated deformation, and could be ignored during the qualitative analysis of the deformation. As the plastic deformation does not cause the volume change of the material, the condition *κ^c^* =*κ_x_^c^* =*κ_y_^c^* =*κ_z_^c^* (isotropic expansion during lithiation in Si) dominates the expansion process of the metasurface. Then, *κ^c^*=1+*βc* could be obtained, and *β=*0.587 is the nondimensional coefficient of compositional expansion and *c* is the normalized nominal Li concentration in Si. Since the film is constrained by the substrate, the plane strain condition is satisfied in the planes perpendicular to the surface of the film. Thus, the plastic flow and lithiation expansion inside the film could cause *κ_x_*=*κ_y_=*0 and *κ_z_=*(1+*βc*)^3^, which means that the Li insertion will mainly increase the thickness of the film and the in-plane deformation could be ignored at the region far from the film edge.

For the mass diffusion part, the chemical potential per mole is given as *μ=RTln(c/(1-c))-Ωσ_h_,* where *σ_h_*= (*σ_1_* +*σ_2_* +*σ_3_*)/3 is the hysrostatic stress, *c* is the normalized nominal Li concentration in silicon, *RT* is the product of gas constant *R* and absolute temperature *T*, *Ω* is the volume per lithium atom in the host. The first term is the driving force for Li diffusion related to the concentration gradient, and the second term shows the influence of stress on the chemical potential. The mass flux of Li into silicon is also given in nominal quantities as *J=-(cD/RT) (∂μ/∂x), D* is the diffusivity of Li in Li-Si alloy. The governing equations for the coupled deformation and mass diffusion include mechanical equilibrium and mass conservation law as *∂σ_ij_/∂x_i_=0* and *∂c/∂t +* *∂J /∂x_i_=0,* where *σ_ij_* is the true stress. In order to simulate the coupled process in the continuous electrodes, the finite element analysis (FEA) could be used, and this method are also related to the molecular dynamics (MD) results at the atomic scale [55]. The Li_x_Si material have been widely studied in the area of LIB. The detailed phase diagram could be found in the reference [1], and the relations of voltages and Li proportion is smooth and continuous of the amorphous Li_x_Si. The quantitatively experimental diffusivity results of amorphous Li_x_Si could be found in the reference [2]. The results show that the diffusion rate of Li-ions in Li_x_Si is much faster than that in the pristine Si. Thus, the rate determining step of the lithiation process is located at the lithiation front inner the film and the top Li_x_Si region will not influence the further diffusion of Li-ions.

**Supporting Figures**


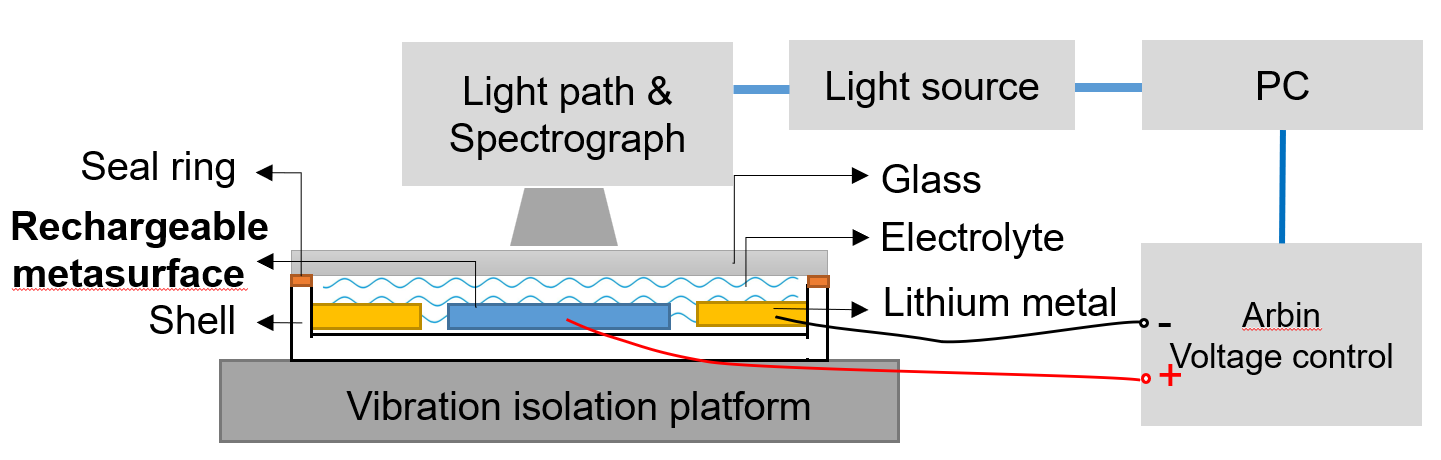


**Figure S1.** Schematic diagram of the in-situ microscopy system. The system includes a home-made in-situ electrochemical cell, the voltage control devices, and a home-built microscopy system. The home-made in-situ electrochemical cell includes the rechargeable metasurface, the reference electrode, the shell, and the optical window. The cell was filled by the electrolyte, and the electrochemical load was conducted by the voltage control devices. The microscopy system includes an imaging microscope and a spectroscopic system.


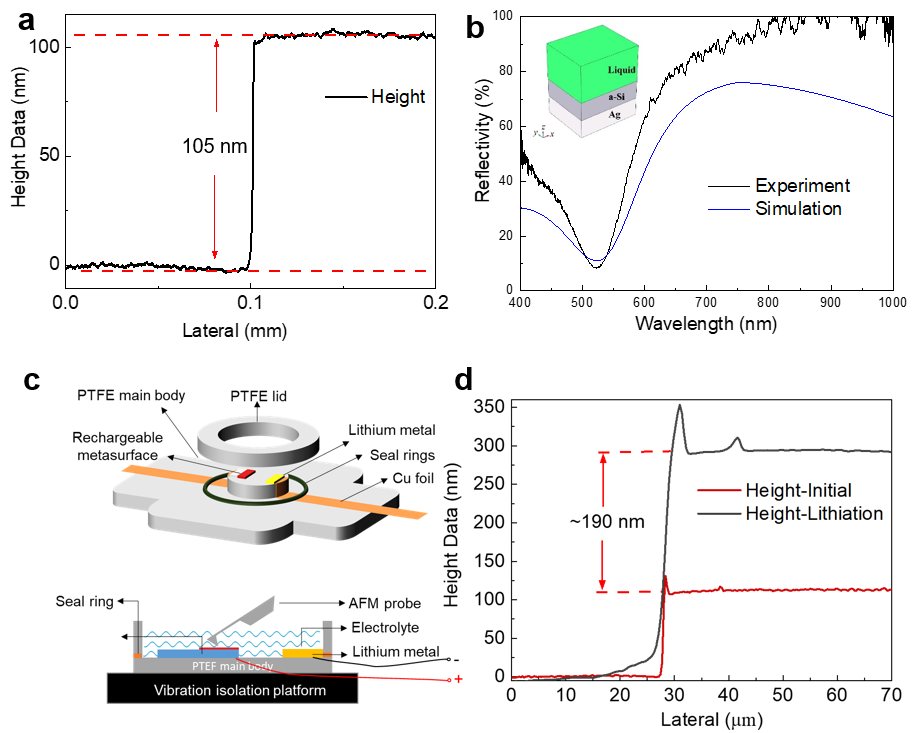


**Figure S2.** Characteristics of a single-layer Si film. (a) Thickness of the Si film measured by a step profiler. (b) Simulated (blue line) and measured (black line) spectra of the initial 105-nm Si film. The inset shows the geometric design in the simulation. (c) Schematic diagram of the in-situ battery and the atomic force microscope (AFM) probe. The counter electrode is lithium metal in this battery. The battery system for the in-situ AFM characterization mainly includes the polytetrafluoroethylene (PTFE) main body and lid, the seal rings and the lithium metal counter electrode. The Cu foil is used as the wire to connect the electrode to the electrochemical loading system. In order to guarantee the stable environment of the open experimental device, the AFM equipment and the in-situ battery were placed in the glovebox (Mbraun Inc.) filled with high purity argon gas. (d) The thickness of the metasurface measured by the AFM before and after lithiation process. The tiny sharp peaks near the step edge are induced by the abrupt movement inertia of the AFM tips and should be treated as measurements errors. As a result, in this work, the average height at the flat region is used to evaluate the thickness evolution of the electrode. The initial thickness of the Si film measured by AFM is about 110 nm which is consistent with the result measured by a step profiler in Figure S2. After the lithiation process, the film thickness increases to around 300 nm, which means the thickness of the film is increased by around 190 nm during the phase change from Si to Li_x_Si. Those in-situ deformation characterization quantitatively reveals the compositional and mechanical regulation process of the metasurface.


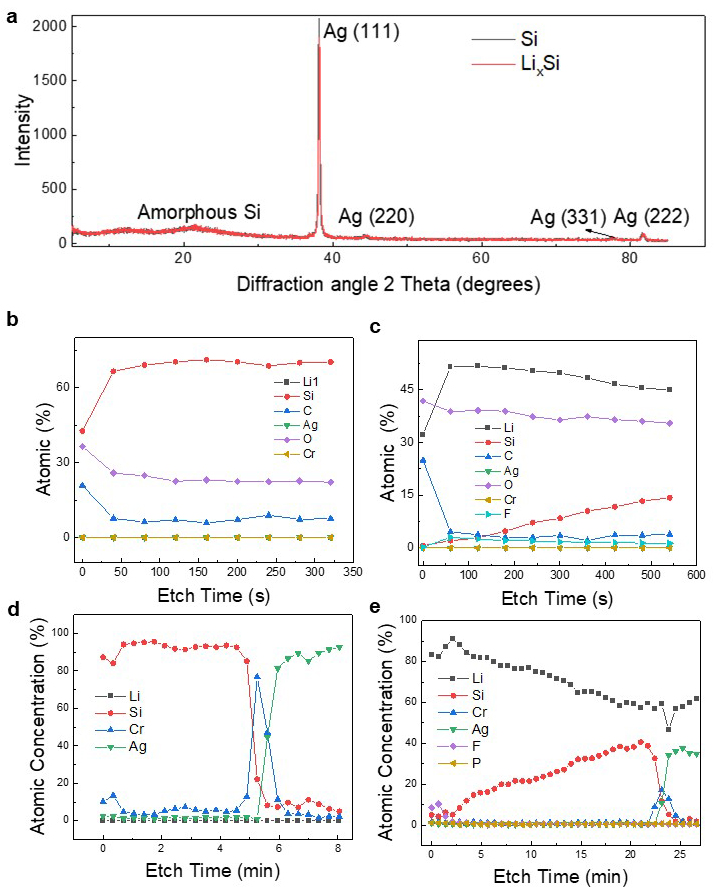


**Figure S3.** (a) The XRD patterns of the electrode before and after lithiation. The XRD (SmartLab, Rigaku Corporation) results confirm that the silicon film is amorphous before and after lithiation. (b and c) The XPS depth-profile experimental results of electrode before and after lithiation. The XPS (ESCALAB Xi^+^, ThermoFisher Scientific) elemental analysis and the depth-profile experimental results show that the Li element has been inserted into the Si electrode. Depth profiling was accomplished by using the instrument’s Ar^+^ ion source operated at 2 keV over a 1 × 1 mm area, and the etch rate was 30 nm/min for SiO_2_. (d and e) The Li distribution throughout the surface to the Ag substrate was characterized by the Auger electron spectroscopy (AES, PHI700 ULVAC), the pressure in the system was less than 3.9×10^-9^ Torr, and primary electron beam energy was 10 keV. Depth profiling was accomplished by using the instrument’s Ar^+^ ion source operated at 2 keV over a 1 × 1 mm area, and the etch rate was 14 nm/min for SiO_2_. As shown in Figure S3d, only the Si elements exist from the surface to the Cr adhesion layer. After the lithiation (turn off) process, the Li elements could be found all over the Si layer, and the Li concentration at the bottom is a little lower than the Li concentration around the surface, from which the Li flux comes. However, the Li concentration can reach around 50% even in the bottom of the Si layer, which is consistent with the XPS results. Those results reveal that the entire Si thin film was lithiated during the lithiation process.


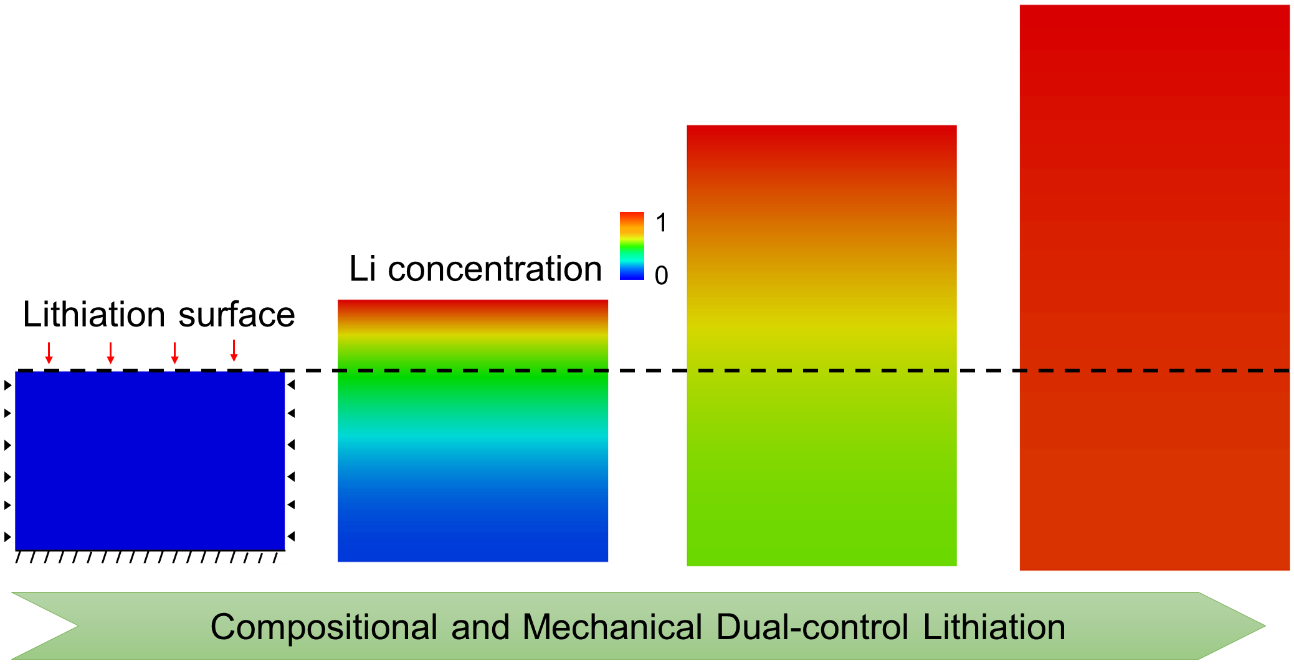


**Figure S4.** The geometric model for finite element analysis (FEA) under the deformation and lithiation process during the coupled process. In the FEA model the bottom of Si film is fixed, and the Li fluxes are loaded on the top surfaces. The dynamics of Li^+^ and Si could be revealed, and the Li^+^ are inserted into the Si electrodes under the electrochemical loading. The electrode swells during the Li^+^ diffusion process.


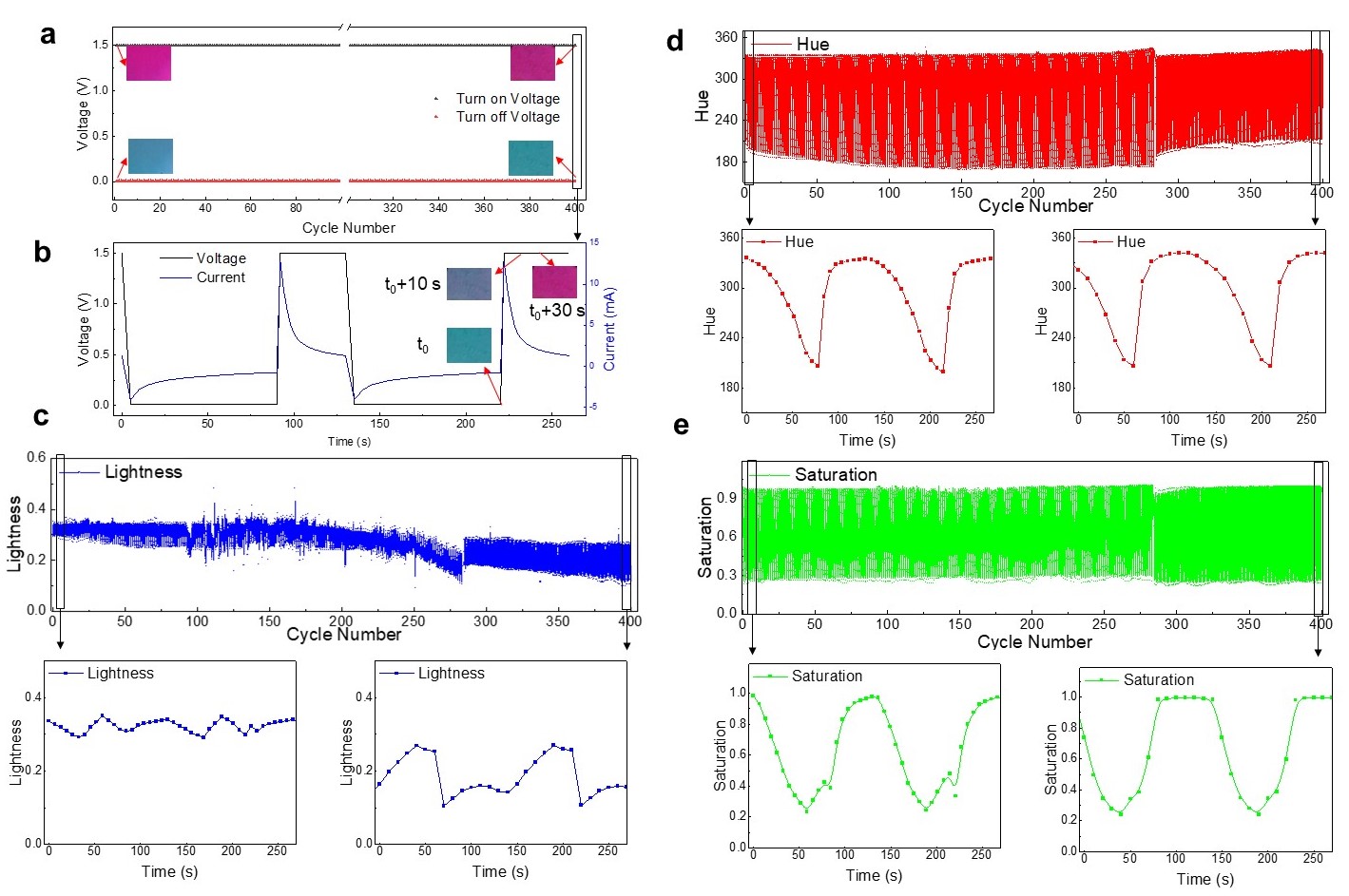


**Figure S5.** (a) The cyclic test of the metasurface. The fast cyclic tests were conducted under the constant voltage control. The cut off voltage of the ‘on’ and ‘off’ states are set as 1.5 V and 0.01 V, respectively. The result shows the color stabilities of the metasurface after 400 cycles, in which the colors of the ‘on’ and ‘off’ states at 400th cycle are consistent with that of the first cycle. (b) The cycle details of 399th-400th cycles, including the Voltage and Current evolution of the system, and the time-resolved color change of the metasurface. The color regulation can be accomplished in 30 s, and the colors are also consistent with the results in Figure 2. The corresponding time-resolved spectra are in correlation with the in-situ spectral evolution in Figure 2a. Different control methods can be used to regulate the metasurface for different purposes. The constant current control can help us obtain more details of the compositional and mechanical dual-altered mechanism, and the constant voltage control with self-adapting current loading can significantly decrease the transfer time of the metasurface. (c-e) The HSL (Hue, Saturation and Lightness represent the color, the intensity or purity of a hue and the relative degree of black or white, respectively) results of the cyclic test. HSL is one of the main ways to represent color values. From the results, the values of Hue and Saturation is more stable than the Lightness during the long cyclic test, and the Lightness of the metasurface has been decreased due to the degradation of the electrode. The variation of the data around 280th cycle is resulted from the resume of the test after optimize the circuit from the LIB to the charge-discharge system.


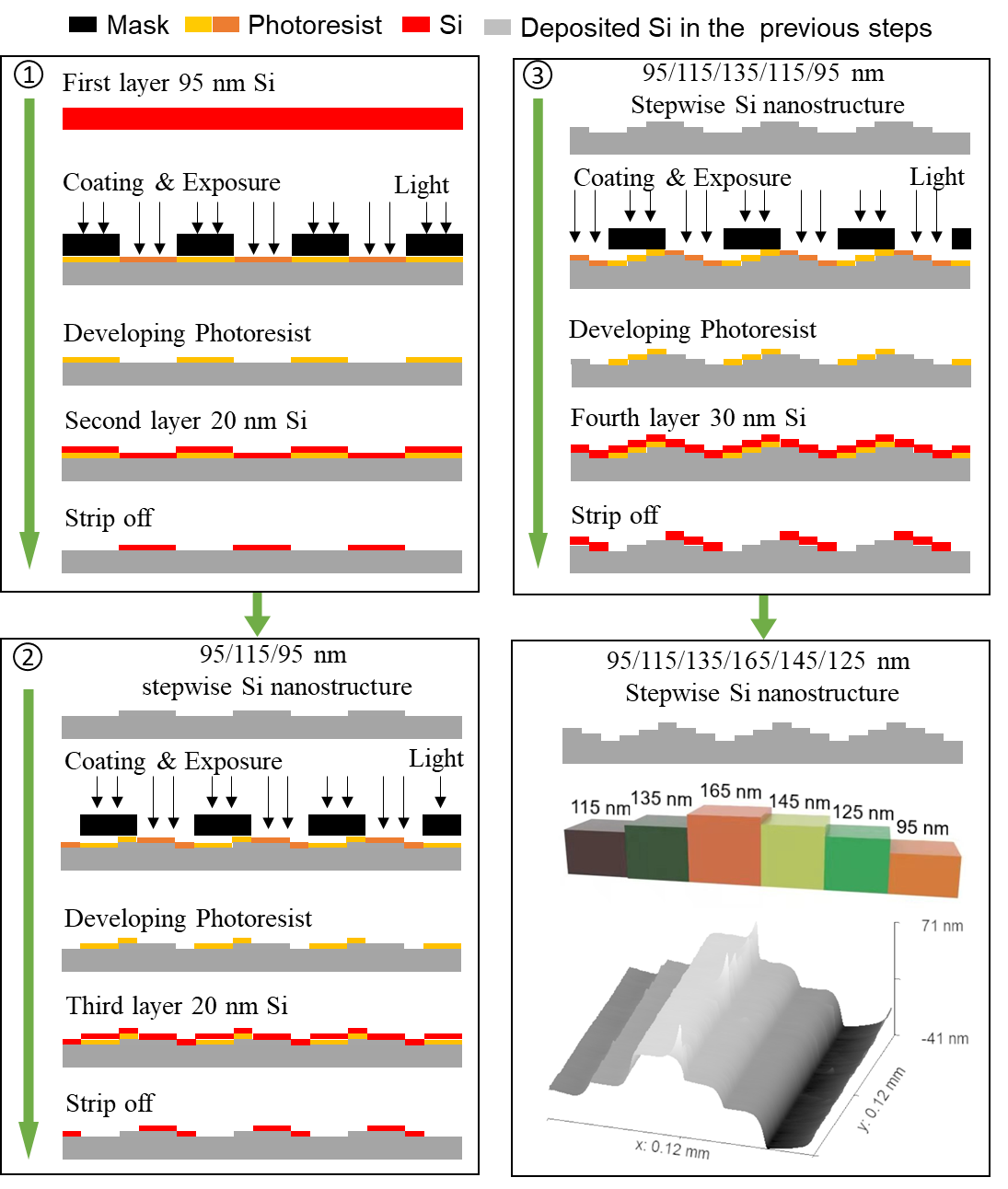


**Figure S6.** Fabrication scheme of the stepwise Si ribbons with various thickness. The Schematic picture of three-cycle photoetching and magnetron sputtering deposition processes, and the three-dimensional morphology of the metasurface measured by the AFM (right-bottom). Only one mask was used during the three-time photoetching. The period of the grilling is 120 µm, and the width of the exposure area is 60 µm. The first layer is ~95-nm-thick Si. The second layer ~20-nm-thick Si was deposited after a standard photoetching process, and the 95/115/95 nm stepwise Si layer was obtained after the lift-off process. Then the same procedures were repeat twice, and the mask was right shifted by 20 µm during each followed photoetching process. Finally, we got the 90/115/135/165/145/125 nm stepwise Si ribbons.


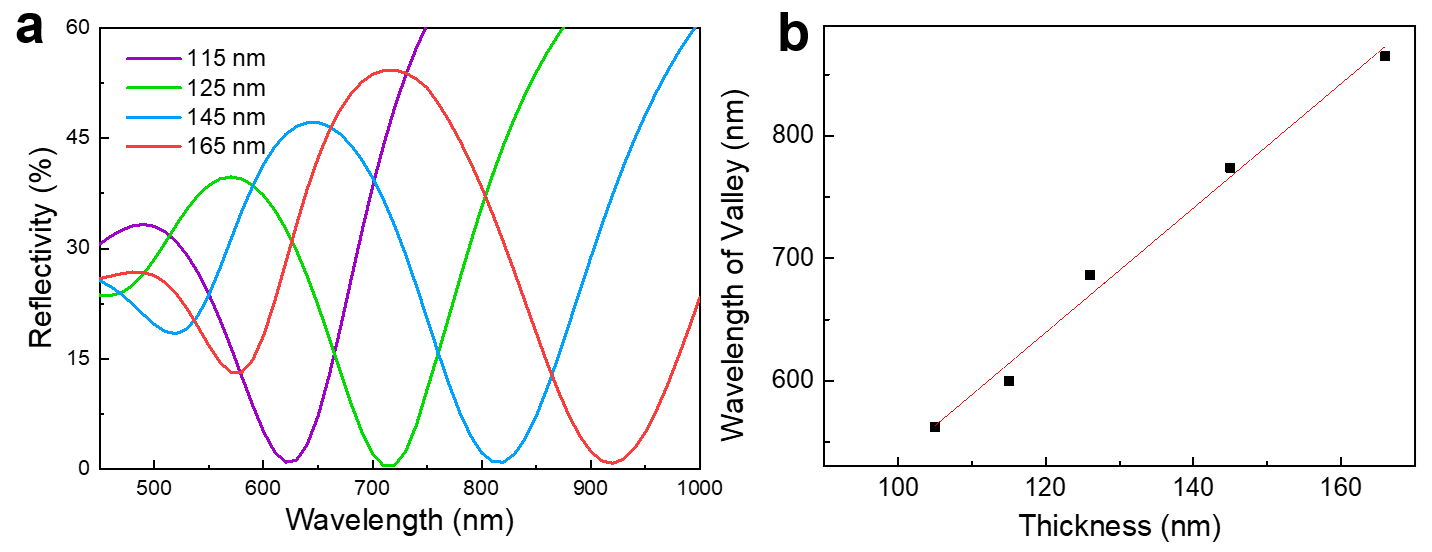


**Figure S7.** Optical properties of Si films with various thickness. (a) Simulated spectra of the Si films with different thickness, agreeing well the experimental measurements in Figure 2c. (b) Relation between the Si film thickness and the spectral dip wavelength.


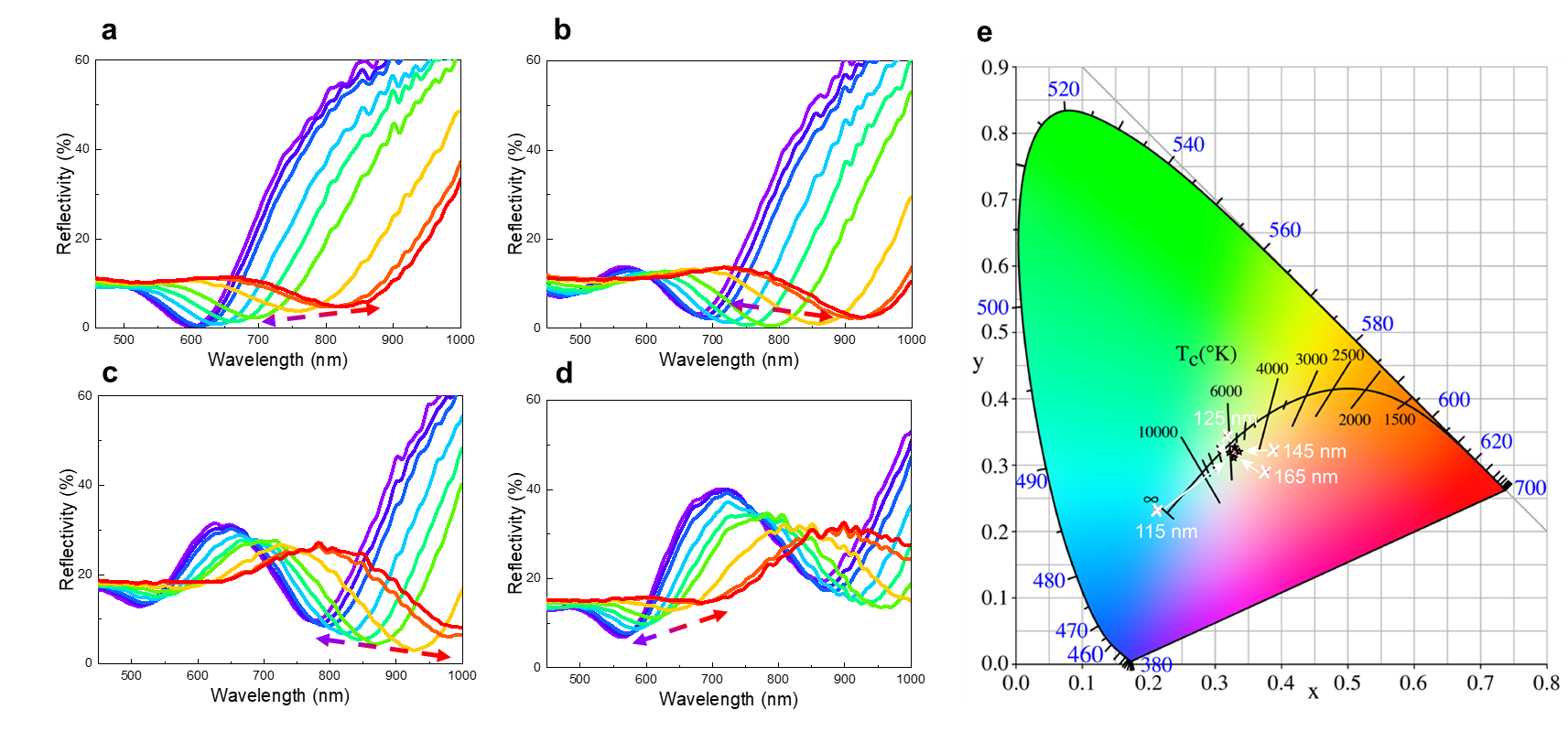


**Figure S8.** As measured spectral evolution and CIE 1931 chromaticity diagram of the Si films with different thickness during lithiation. (a-d) correspond to the spectrum of Si films with thickness of 115, 125, 145, and 165 nm, respectively. From blue to red lines, the voltage changes from 1.5 to 0.01 V. (e) CIE xy chromaticity coordinates of the measured reflection spectra of Si ribbons with different thickness in Figure 2c,d under the color “on” state (white markers) and “off” state (black markers).


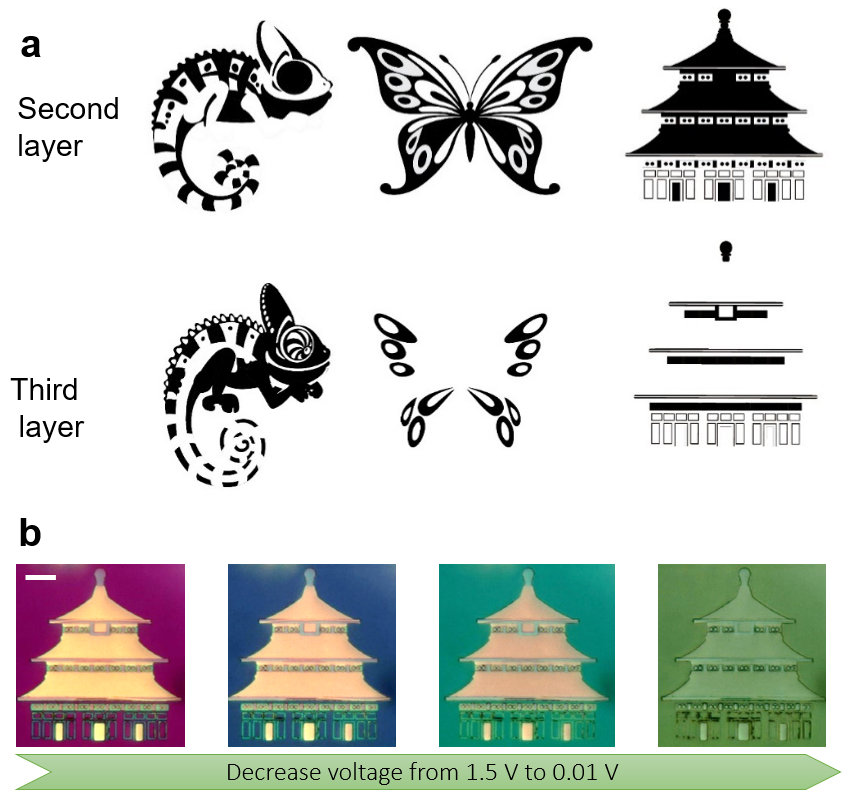


**Figure S9.** Detailed construction of thin-film color displays. (a) The first and second layer used during the preparation of lithography. The first layer is ~80-nm-thick Si film. By using laser direct writing photoetching (DWL66+, Heidelberg) and magnetron sputtering deposition processes, ~40-nm Si and ~20-nm Si patterns was deposited according to the second and third layer, respectively. Thus, ~60-nm Si could be deposited at the overlapped area of the second and the third layer, which results in a ‘chameleon’, ‘butterfly’ and ‘Temple of Heaven’ patterns with Si of four thickness (about 80, 100, 120, and 140 nm). (b) The color change of ‘Temple of Heaven’ during the lithiation process. Scale bar: 50 μm.


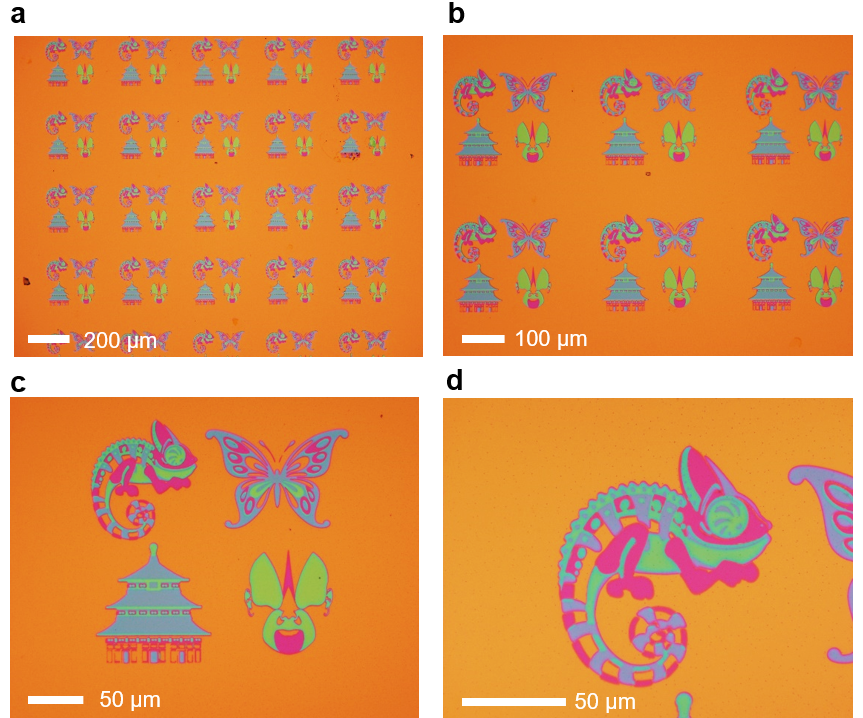


**Figure S10.** Large-area color displays of the as-fabricated nanofilm. The dark-field images are captured by a microscope (BX51M, OLYMPUS, light source: U-LH100HG), exhibiting the ability of preparing large-area color display devices base on the ECM film. The unit size of each four patterns is 200 μm×200 μm. The colors of the patterns under normal incidence will transform to the stable reversible initial colors in the first image of Figure 3b after electrochemically activated by a pre-charge-discharge cycle. This activation is necessary due to the formation of irreversible solid electrolyte interphase (SEI) at the surface of Si during the first electrochemical cycles. Nevertheless, the SEI film is very stable during the subsequent electrochemical cycles [3,4].


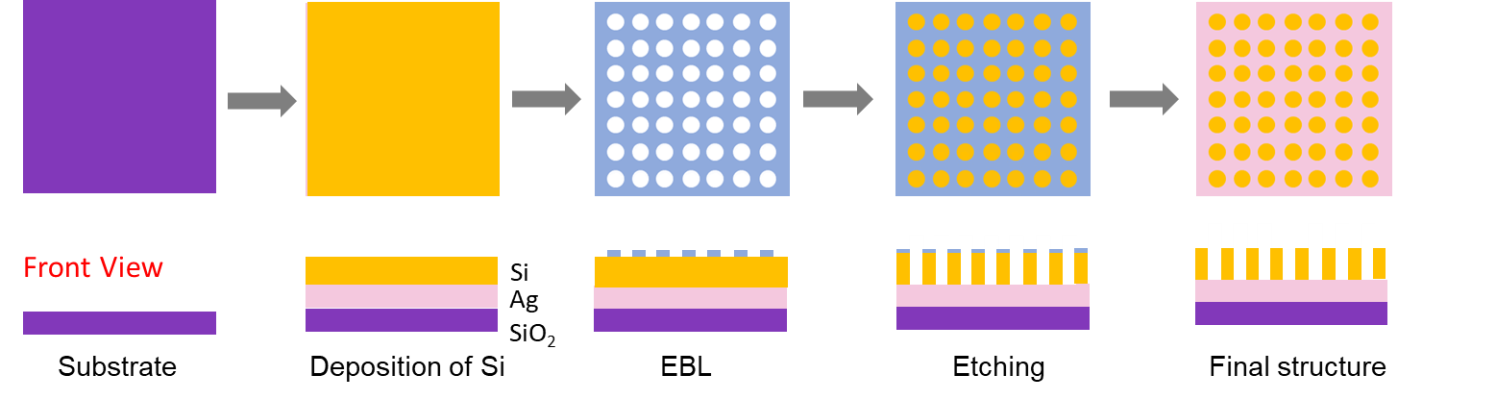


**Figure S11.** Flowchart of the EBL processes for the high-resolution fabrication of isolated nanostructures.


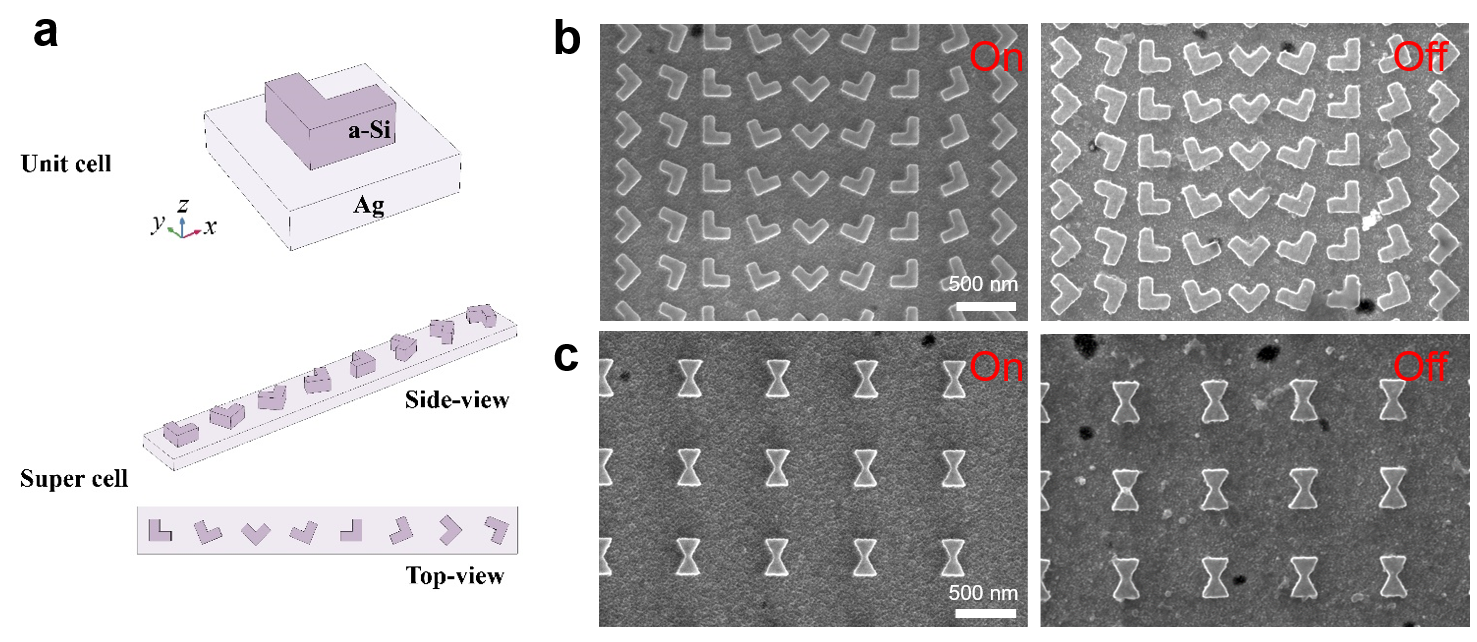


**Figure S12.** (a) Schematic of L-shaped nanostructure. (b) L-shaped metasurface with period of 400 nm before (right) and after (left) the lithiation. (c) The bowtie nanostructures with overlapped tips before and after the lithiation.

**
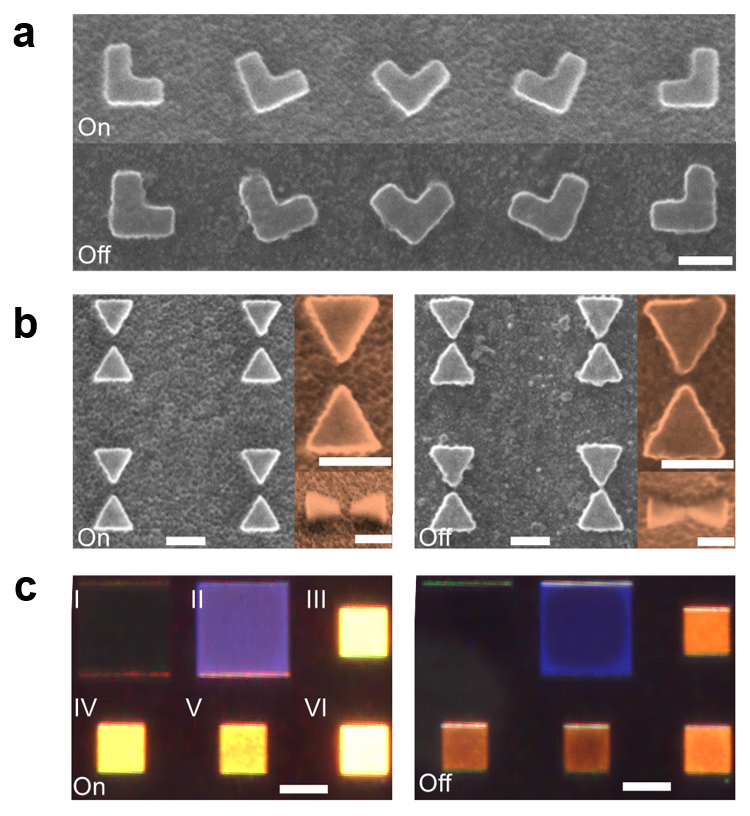
**

**Figure S13.** Reconfigurable optical nanostructures. (a and b) SEM images of typical L-shaped (*p*=500 nm) and bowtie nanostructures (*p*=500 nm) before and after lithiation. Scale bar: 200 nm. The geometric changes induced by the electrochemical process are clearly seen. (c) Optical microscopy images of various metasurfaces at the On (left) and Off (right) states. The incident and reflection angles are *α*=60° and *β*=0°, respectively. Structures II and IV correspond to the nanostructures shown in Figure S13a,b, respectively. I, III, V, VI correspond to structures of different sizes and nanogaps (Figure S12b,c), among which the touched bowtie nanostructures show the highest diffraction intensity. Scale bar: 50 μm.

**
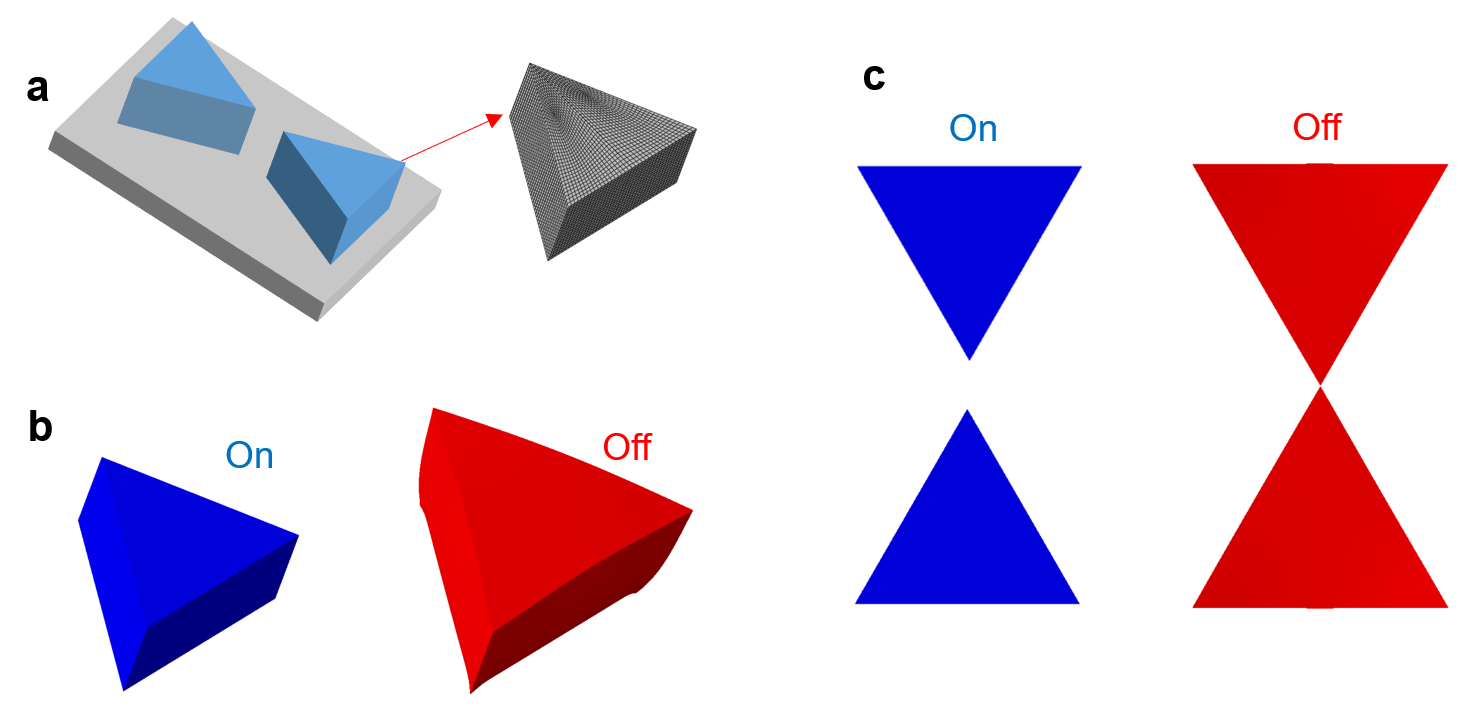
**

**Figure S14.** (a) Geometric model and the mesh for finite element analysis (FEA). In the FEA model, the bottom of Si electrode was subjected to vanishing displacement boundary condition, and the Li fluxes were prescribed on the top and side surfaces of the bowtie nanostructures. For silicon, a classical bilinear elastic-plastic mode was used, and the elastic module is *E* =130 GPa, the Poisson ratio *ν*=0.3, the yield stress *σ*_Y_ = 0.26 MPa, and the coefficient of compositional expansion=0.4 during lithiation process. (b) 3D deformation and (c) top view of the bowtie nanostructures at On and Off state. The detailed simulation method and the parameters of Li_x_Si used during FEA could be found in our previous work [5].


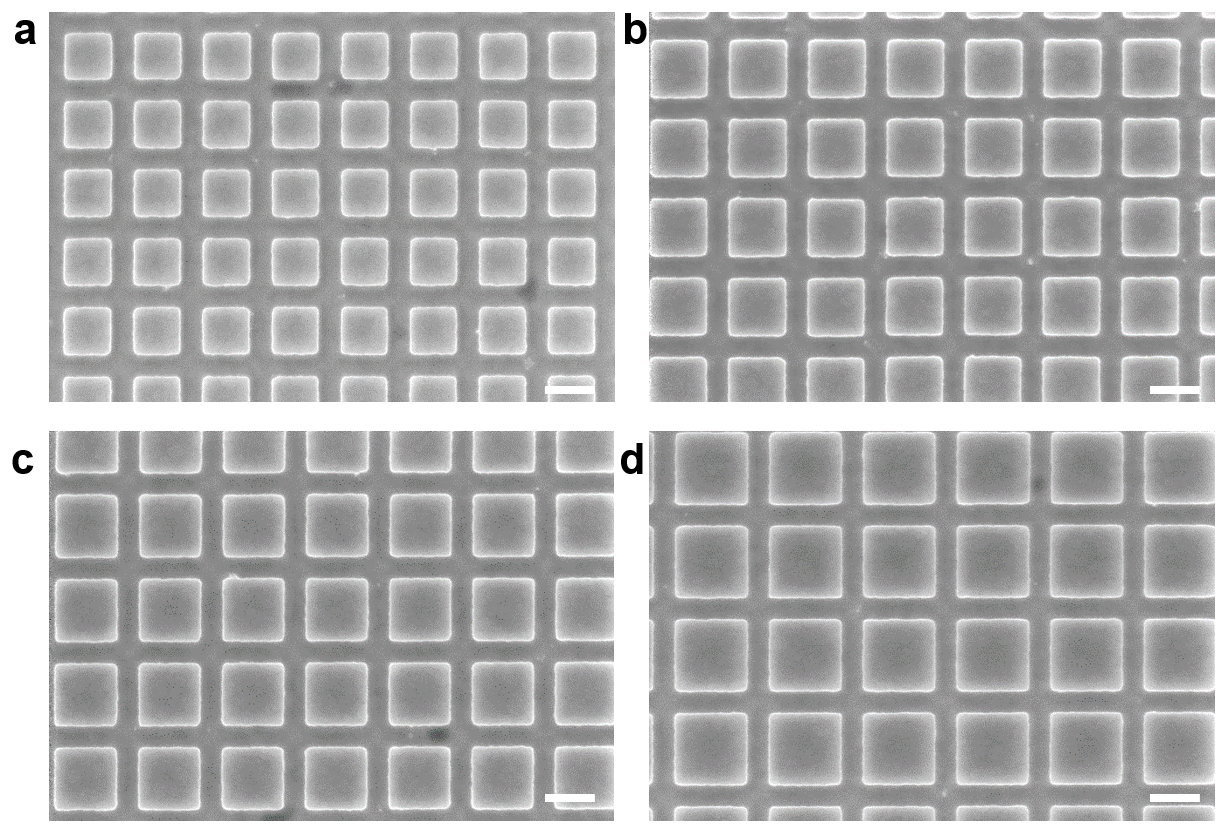


**Figure S15.** SEM images of square arrays. (a-d) are the square array with length of ~180, ~220, ~240, and ~280 nm, respectively, under the same period of 380 nm. Scale bar: 200 nm.


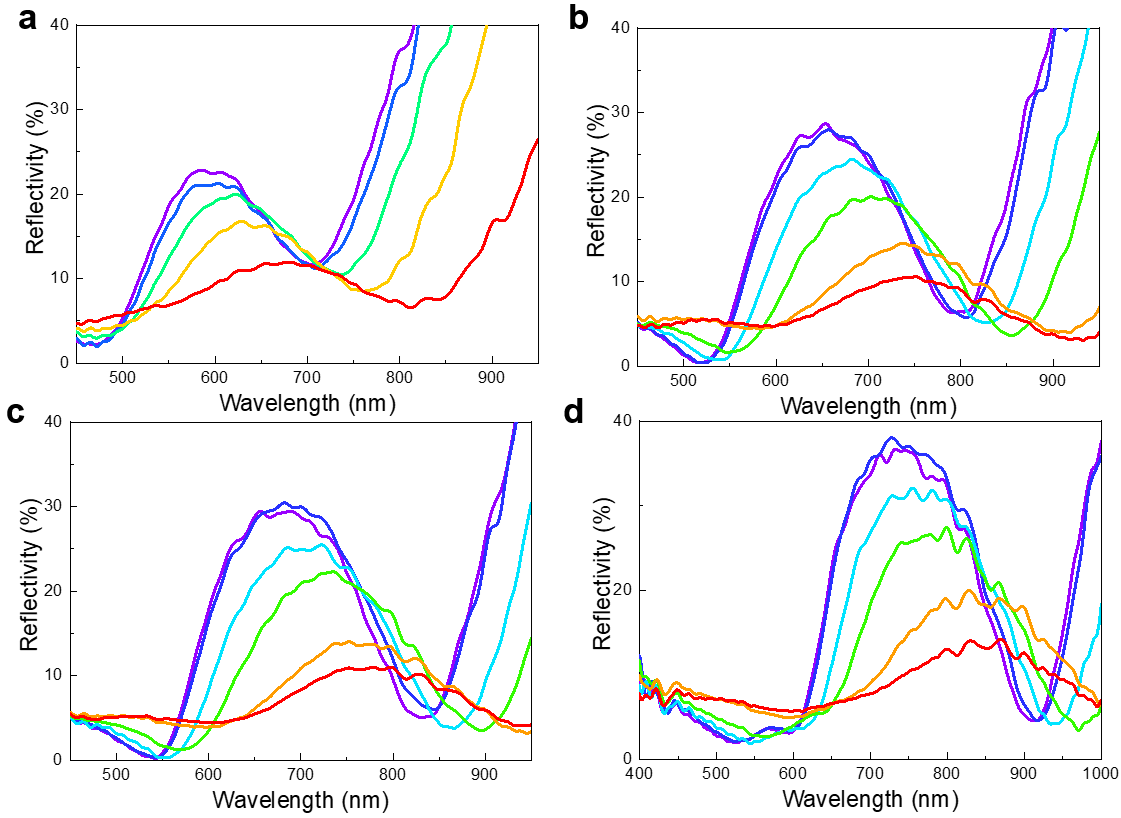


**Figure S16.** As-measured continuous spectral evolution of the square arrays shown in Figure S15. From blue to red lines, the voltage changes from 1.5 to 0.01 V.


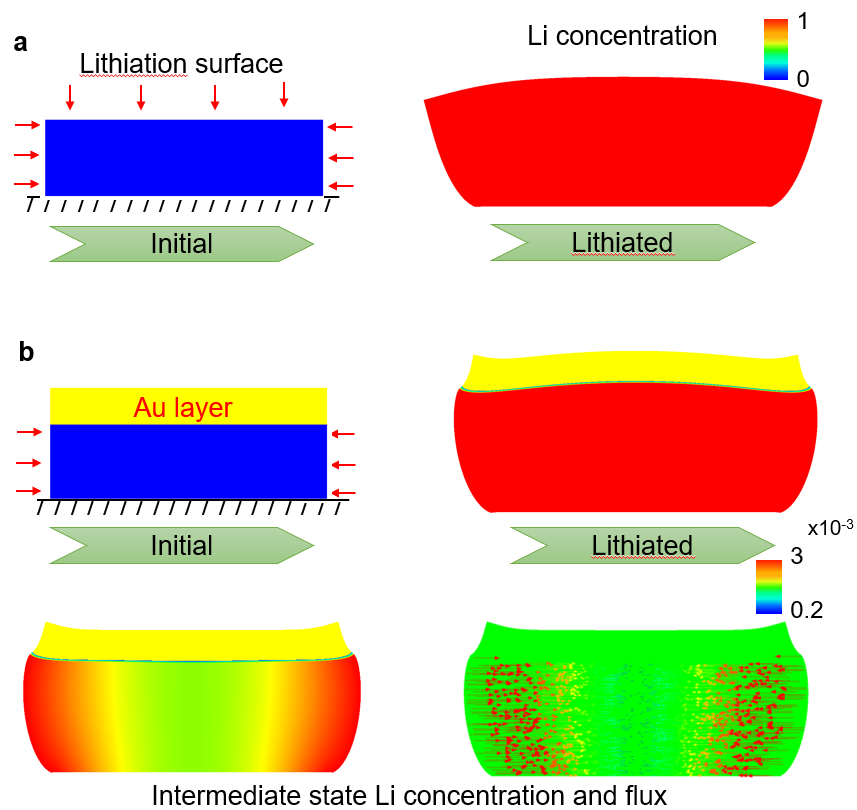


**Figure S17.** The simulation results of mechanical and compositional dual process of structures with (a) and without top Au layer (b). Comparing with the structure without top Au layer, the Au will restrict the lithiation path at the top surface. In such a case, the side surfaces offer the channel for Li. The result shows that the Li inserted into the Si from the side surfaces, and the Si can be fully lithiated. Although the Au indeed constrains some deformation of the electrodes at the horizontal direction, the nanostructures can still swell at the vertical direction which affect the key structural parameter of the FP cavity.


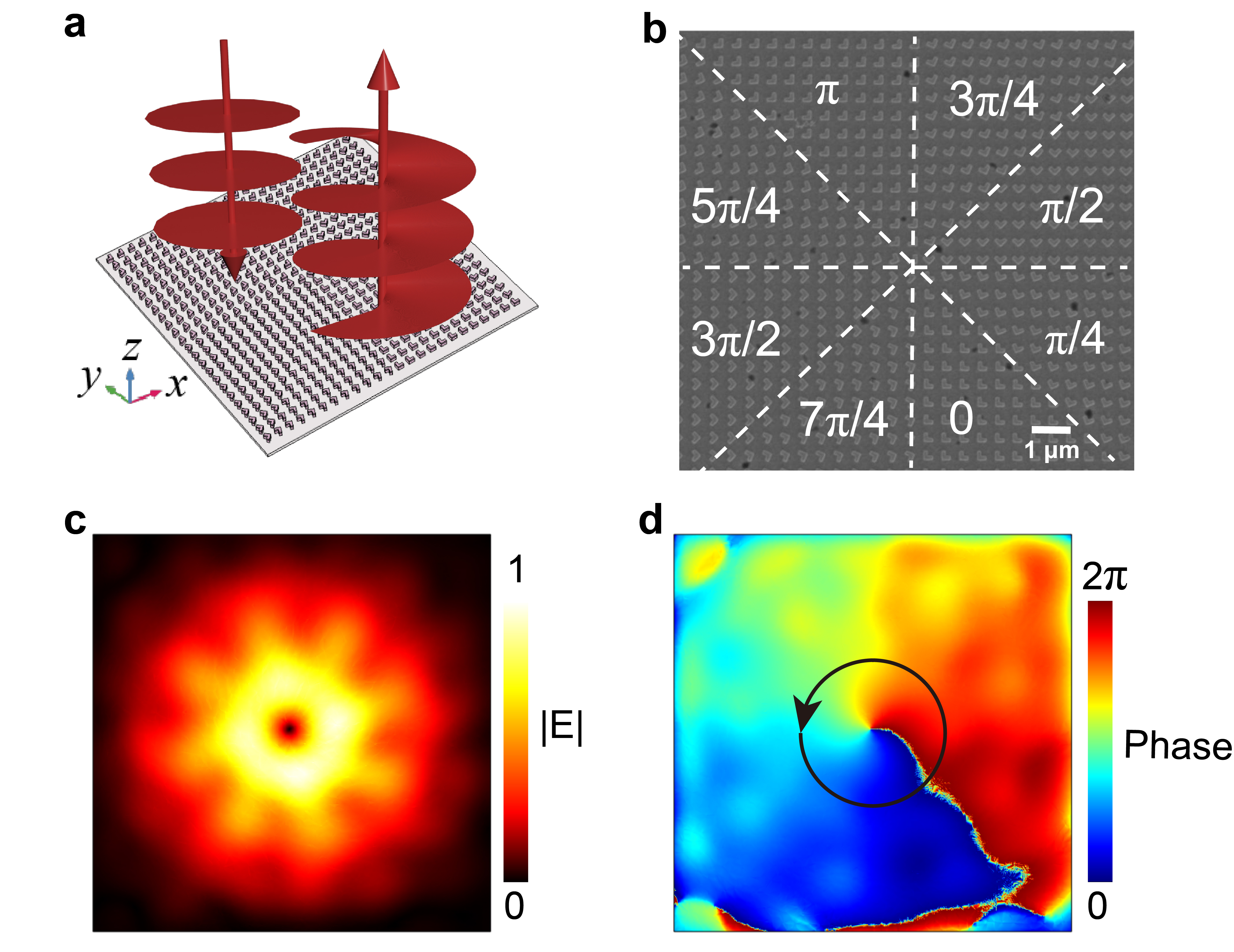


**Figure S18.** A proposal for an optical vortex converter based on L-shaped nanostructures. (a) Schematic diagram of generating vortex light via incident circularly polarized light. (b) Partial SEM image of the optical vortex converter based on the L-shaped nanostructures fabricated with the method in this work. The width, length and periodictiy of the L-shaped nanostructures are 80, 200, and 400 nm, respectively. Scale bar: 1 μm. (c) Simulated normalized near-field amplitude distribution under circularly polarized light incidence of Gaussian beam in Figure S18b. (d) Simulated near-field phase distribution under circularly polarized light incidence of Gaussian beam in Figure S18b. The results in Figure S18c,d clearly show the existence of optical vortex with topological charge of +1. The charging and discharging processes can significantly change the structure morphology and their spacing, which naturally leads to the change in the structural scattering phase distribution in Figure S18b. Thus, it can be expected that the optical vortex modulations could be potentially achieved via charging and discharging to different phase regions in future studies.


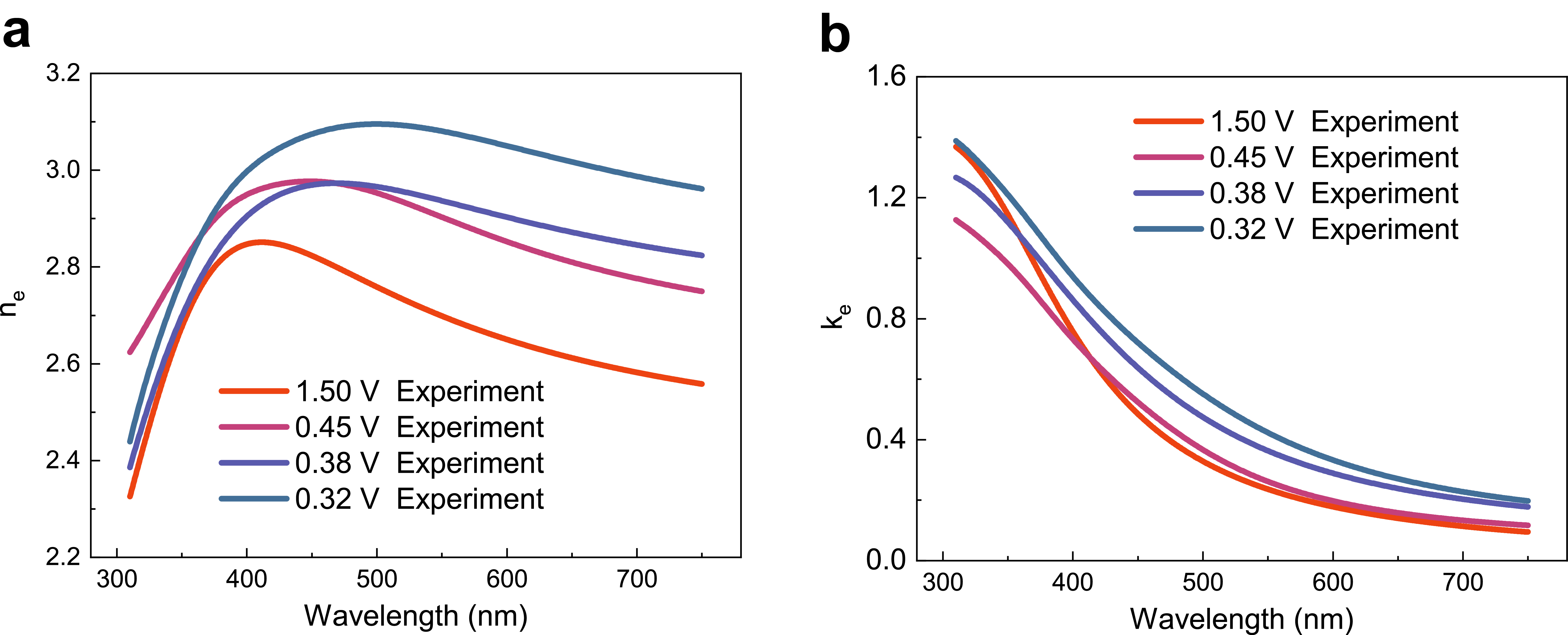


**Figure S19.** Refractive index data originated from our previous work [56]. (a) Refractive index, and (b) absorptivity, calculated from the spectroscopic ellipsometry results at different voltage stages. There is an increase of the effective refractive index above 450 nm upon lithiation, while the effective absorptivity increases slightly upon lithiation.

**Legends for Movies**

**Movie S1 (separate file).** The continuous color changes of 105-nm-thick Si film during electrochemical cycles. The basic color evolution of the single thick Si film, the corresponding spectral evolutions are shown in Figure 2a. This basic process provides great feasibility for dynamic color printing by engineering the film thickness.

**Movie S2 (separate file).** The continuous color changes of a stepwise Si ribbon structure during electrochemical cycles. A stepwise Si ribbon structure is designed and fabricated by repeated ultraviolet exposure and film deposition processes. Six different colors from Si ribbons of various thickness were clearly observed in the experiments. The multiple colors can be simultaneously and reversibly tuned by the cyclical lithiation and delithiation processes, the corresponding spectral evolutions are shown in Figure 2c,d.

**Movie S3 (separate file).** The continuous color changes of a chameleon pattern during electrochemical cycles. A chameleon pattern with Si layers of four thickness (80, 100, 120, and 140 nm). Such a fabrication method is very desirable for large-scale color printing. The reversible colorization and decolorization of chameleon and butterfly can be readily achieved by simply controlling the external voltage within 1.5 V.

**Movie S4 (separate file).** The continuous color changes of a butterfly pattern during electrochemical cycles.

References

1. Kwai S C, Wu-Wei L and Candace K C. First-principles studies of the lithiation and delithiation paths in Si. *J Phys Chem C* 2019, **123**: 22775-86.

2. Luize Scalco de V, Rong X and Kejie Z. Quantitative spatiotemporal Li profiling using nanoindentation. *J Mech Phys Solids* 2020, **144**:104102.

3. Peled E and Menkin S. Review-SEI: Past, present and future. *J Electrochem Soc* 2017; **164**: A1703-19.

4. Chen HS, Han Y and Yang L *et al*. A method for analyzing two-dimensional lithium ion concentration in the nano silicon films. *Appl Phys Lett* 2019; **115**: 264102.

5. Yang L, Chen HS and Jiang H *et al*. Failure mechanisms of 2D silicon film anodes: in situ observations and simulations on crack evolution. *Chem Commun* 2018; **54**: 3997.
